# Exploring the personal and professional factors associated with student evaluations of tenure-track faculty

**DOI:** 10.1371/journal.pone.0233515

**Published:** 2020-06-03

**Authors:** Dakota Murray, Clara Boothby, Huimeng Zhao, Vanessa Minik, Nicolas Bérubé, Vincent Larivière, Cassidy R. Sugimoto

**Affiliations:** 1 School of Informatics, Computing, and Engineering, Indiana University, Bloomington, Indiana, United States of America; 2 Department of Sociology, University of Chicago, Chicago, Illinois, United States of America; 3 École de Bibliothéconomie et des Sciences de l’information, Université de Montréal, Montreal, Quebec, Canada; Universitá degli Studi di Bergamo, ITALY

## Abstract

Tenure-track faculty members in the United States are evaluated on their performance in both research and teaching. In spite of accusations of bias and invalidity, student evaluations of teaching have dominated teaching evaluation at U.S. universities. However, studies on the topic have tended to be limited to particular institutional and disciplinary contexts. Moreover, in spite of the idealistic assumption that research and teaching are mutually beneficial, few studies have examined the link between research performance and student evaluations of teaching. In this study, we conduct a large scale exploratory analysis of the factors associated with student evaluations of teachers, controlling for heterogeneous institutional and disciplinary contexts. We source public student evaluations of teaching from *RateMyProfessor.com* and information regarding career and contemporary research performance indicators from the company *Academic Analytics*. The factors most associated with higher student ratings were the attractiveness of the faculty and the student’s interest in the class; the factors most associated with lower student ratings were course difficulty and whether student comments mentioned an accent or a teaching assistant. Moreover, faculty tended to be rated more highly when they were young, male, White, in the Humanities, and held a rank of full professor. We observed little to no evidence of any relationship, positive or negative, between student evaluations of teaching and research performance. These results shed light on what factors relate to student evaluations of teaching across diverse contexts and contribute to the continuing discussion teaching evaluation and faculty assessment.

## Introduction

Performance indicators have come to dominate faculty evaluations of teaching and research at universities in the United States, raising concerns over their consequences [1]. One of the most prominent indicators for teaching are student evaluations of teaching (SETs), in which students anonymously score and comment on their course instructors for the purpose of evaluation and improvement. However, SETs alone are not sufficient for evaluation of tenure and tenure-track faculty for whom teaching constitutes only a portion of their professional responsibilities. Contemporary research universities are built on the premise that faculty balance research, service to the academic community, and teaching (see Boyer’s model of scholarship [2]). Holistic faculty evaluation requires assessments along each of these dimensions and of the faculty’s ability to balance their commitments. However, quantitative studies of SETs typically have not examined teaching ratings in relation to faculty performance in other professional activities. Studies of SETs are also limited by the difficulty of aggregating data across institutional contexts, which has resulted in a poor understanding of the extent to which SETs depend on institutional and disciplinary factors. There is a need for a large-scale analysis of SETs to provide a more complete understanding of the extent to which these evaluations relate to personal or professional characteristics of teachers, institutional context, and research performance.

Questions of bias in SETs have prompted intense scrutiny and numerous studies on their validity. For example, past research on traditional SETs has identified biases based on gender [3–8], race [6, 9], attractiveness [10], and age [7, 11, 12]. Many have also criticized traditional SETs as invalid measures of teaching quality and student learning [3, 7, 12–17] and warned university administrators against using them for hiring and promotion decisions [18]. In light of these issues, there have been intensifying claims that SETs harm both students and faculty [19] and public calls to stop relying on them for evaluating teaching [20, 21]. In spite of this controversy, SETs have remained one of the most common metrics of teaching performance across a variety of U.S. universities [22]. Given their continued use for hiring and promotion, there remains a need to study the factors contributing to outcomes on SETs.

The *research-teaching nexus* refers to the relationship between time spent doing research, and time spent teaching. The Humboldtian ideal of a university is built on the premise that these tasks are mutually beneficial [23], and many have followed this tradition, positing a strong relationship between research and teaching [24–27]. However, there is a lack of consensus surrounding the presence, extent, and nature of the nexus. While some studies have found evidence of *positive* research-teaching nexus—a mutually-beneficial relationship [28–30], other studies have instead observed a *negative* research-teaching nexus, suggesting that faculty incentive structures encourage research at the expense of teaching quality [31–33]. Conflicting with both the positive and negative nexus hypotheses, a landmark meta-analysis instead suggested a *neutral* research-teaching nexus, observing no evidence of a relationship between research and teaching [34]. Taken together, these studies offer no clear understanding of the research-teaching nexus; moreover, these studies have tended to be small and limited to particular institutional contexts. There remains a pressing need to understand the research-teaching nexus at scale and across institutional contexts.

In this study, we conduct a large-scale exploratory investigation of the extent to which demographic characteristics and research performance relate to SETs for tenured and tenure-track faculty in the United States. We leverage public teaching evaluations from *RateMyProfessor.com*, a public data source of public SETs which, despite criticism [35, 36], has been found to correlate with traditional evaluations [37–40]. We match these teaching evaluations with records from *Academic Analytics*, a research analytics company which provided us with a list of active tenured and tenure-track faculty in the United States, along with indicators of their number of publications, citations, grants, and professional awards. In performing this analysis, we hope to gain a more complete understanding of how individual, classroom, university characteristics, and research performance correlate with university faculty’s teaching evaluations. We also aim to shed light on the research-teaching nexus, the relationship between research and teaching.

## Data and methods

### Academic analytics

*Academic Analytics* is a U.S. based company that sells access to their proprietary dataset of individual-level bibliometric indicators for use by university administrators in the United States and the United Kingdom to assess their departments. This data is derived from a mix of direct cooperation with research institutions and collection from publicly available sources such as institutional websites, CrossRef, and Federal agencies. We maintain a contract with Academic Analytics, through which we are granted a copy of their 2017 data release (AA2017).

The version of AA2017 used in this study contained demographic and bibliometric data for 165,666 tenure and tenure-track faculty at 399 universities and research institutions in the United States. AA2017 contains full names, departmental and institutional affiliations, year of doctoral attainment, and disciplinary classification. The dataset also included bibliometric indicators of recent scholarly performance: indexed publications produced in the previous five years; citations to those publications; grants held in the previous five years; lifetime professional awards won; and books published within the past ten years. Details and definitions of the relevant variables from AA2017 can be found in S1 Table.

### RateMyProfessor.com

*RateMyProfessor.com* is a website offering students at institutions of higher education the opportunity to review their teachers and to read reviews by other students. Founded in 1999, the most recent version of *RateMyProfessor.com* allows students to anonymously review teachers along dimensions of overall quality, level of difficulty, and until recently, “hotness”—a binary rating implicitly associated with physical attractiveness (see S1 Text for discussion of rating types removed from the website). Ratings on *RateMyProfessor.com* have been found to correlate with traditional student-evaluations of teachers (see S1 Text). Students are also encouraged to post comments to elaborate on their experience, and to select from a list of pre-defined “tags” that describe the common characteristics of the teacher and the course. Teachers, courses, and schools are all added to *RateMyProfessor.com* by users, and so the presence of any individual depends on the effort of students. Although the website has passed through many iterations, these core features have remained roughly consistent over time. *RateMyProfessor.com* remains one of the only and most popular large-scale, publicly available source of students’ evaluations of teachers, boasting “…more than 19 million ratings, 1.7 million professors and over 7,500 schools” [41]. We collected these data in January of 2018. Details and definitions of relevant variables from this data can be found in S2 and S3 Tables.

### Disciplinary aggregation

The AA2017 dataset used a hierarchical three-tiered disciplinary taxonomy, with the most granular tier consisting of 171 distinct classifications that were applied based on each individual’s departmental affiliation. When an individual held multiple affiliations or when a program was classified as more than one discipline, *Academic Analytics* duplicated their entire record, changing only their disciplinary classification. Thus, while there were 165,666 unique tenure and tenure track faculty represented in in AA2017, 42,500 of these individuals had at least one duplicate record, which resulted in 225,877 total records.

To streamline the large variety of AA disciplinary classifications, we manually mapped each of the AA2017 171 detailed classifications to one of the five NSF classifications of research discipline: *“Natural Sciences”*, *“Medical Sciences”*, *“Social Sciences”*, *“Humanities”*, and *“Engineering”*. After we applied these broad disciplinary classifications, 16,254 individuals had duplicate records with distinct NSF classifications, compared to the 42,500 with distinct *Academic Analytics* classifications.

### Processing research indicators

We added a new research indicator for each individual, *Publication Count*, which we defined as the sum of their indexed conference proceedings, book publications, and article publications; this combined indicator simplifies analysis, and captures the range of publications types that have distinct disciplinary distributions [42] (see distributions in S1 Fig). The final indicators included the number of recent publications (5 years for articles and conferences, 10 for books), the number of citations to those recent publications, the number of grant dollars currently held, and the number of lifetime professional awards held by the individual. We field-normalized each AA2017 research indicator by the mean across the 171 granular disciplinary categories. This was performed for each record, normalizing by the mean of that record’s associated granular discipline. For example, if an individual published ten times within the past five years, and had two records, one for discipline A, with a field-mean of 5 publications, and one for discipline B with field-mean of 15 publications, then that individual’s records would have field-normalized scores of 2.0 and 0.667, respectively.

We also created discretized versions of each continuous field-normalized indicator of research performance. We binned each research indicator into an ordered factor containing a value of “None”, “Moderate”, or “High”. A classification of “None” meant that a count of zero is reported for that indicator. “Moderate” meant that the reported count is between the 1st and 90^th^ percentile (inclusive) for that research indicator, calculated on the population of individuals who have a count greater than one. “High” meant that the reported count was above the 90^th^ percentile of those with a count of at least one for that indicator. We performed this discretization because each field-normalized indicator is strongly zero-inflated and right-skewed (see the log-log distribution of indicators in S1 Fig); these categories mitigated the impact of outliers and allowed for a clearer comparison between those with and without recent research activity.

### Record matching

After the above pre-processing steps, we attempting to match records between the AA2017 and RMP2018 datasets. For each individual in AA2017, we attempted to find a likely match within RMP2018. After extensive experimentation and parameter tuning we settled on using Jaro-Winkler string distance [43–45] as the measure of distance between records. This measure offers flexibility to handle minor variation in instructor and department names. Distance between two strings is based on the number of character matches that occur in similar indexes in both strings, and includes a penalty factor that penalizes strings that have a mismatch within the first few characters. Given that this measure prioritizes matches early in the string, we format match strings for records in AA2017 and RMP2018 as follows,

$$\begin{array}{c} \left[\text{LAST}\;\text{NAME}]\;[\text{MIDDLE}\;\text{INITIAL}]\;[\text{FIRST}\;\text{NAME}]\;[\text{PROGRAM}\;\text{AFFILIATION}\right] \end{array}$$

where [*PROGRAM AFFILIATION*] is the “Program Name” variable in AA2017 and the “Department” variable in RMP (see S2 and S3 Tables for descriptions of these variables). Using this format, *Jaro-Winkler* distance will tend to enforce strict similarity between last names while allowing for some increased variation in first names and department names. This is especially useful for faculty who use informal nicknames while teaching; for example, an individual in AA2017 with the match string *“Smith Robert Applied Mathematics”* results in a relatively high similarity score with an individual from RMP2018 with the name *“Smith Bob Applied Mathematics”*.

We calculated pairwise *Jaro-Winkler* string distances between the match strings for each individual in AA2017 and each profile from RMP2018. If the largest similarity metric between a record from AA2017 and any profile on RMP was lower than 0.1, then we excluded that individual from the final dataset. If at least one RMP profile has a similarity score above the threshold, then the most similar profile was selected as a match. This process resulted in 47,509 matches between individuals in AA2017 and RMP, representing 34.5 percent of AA2017 records, and 3.0 percent of all RMP2018 records; this small population of matched RMP2018 records is expected because *RateMyProfessor.com* included non-tenured/non-tenure track faculty, faculty who are no longer active, and faculty from countries not represented in our version of AA2017.

A discussion of the representativeness and potential biases in our matching process can be found in S1 Text for AA2017 and RMP2018.

### Gender assignment

We assigned a gender to each record in the matched dataset by comparing the number of masculine and feminine pronouns that appeared in text reviews left on faculty’s profiles on *RateMyProfessor.com*. If the reviews of a profile contained more of one type of gendered pronouns than the square of the other, then we assigned their gender using the gender of the majority pronoun. For example, if one profile’s reviews contained a total of ten masculine pronouns (e.g.: “he”, “him”, “himself”), but only three feminine pronouns (e.g.: “she”, “her”, “herself”), that profile would be assigned a gender of male (10 > 3^2^); however if a profile contained four masculine and three feminine pronouns, then no gender was assigned (3^3^ > 4). Using this method, we assigned a gender of male or female to 99.7 percent of tenure and tenure-track professors in the final matched dataset.

### Race assignment

We infer a race for each individual in our dataset from their surname. We retrieved the dataset of surnames from the US Census, which contains, for each surname, the percentage of individuals having that name that are White, Black, Asian, Hispanic, Native American or Pacific Islander, and two or more races, as determined by the census. We adopt a conservative and course-grained approach to inferring race from these information; An individual in our dataset is assigned as *Likely White* when at least 70 percent of those having the same surname are White. Otherwise, an individual is assigned *Likely Non-White*. When an individual’s surname does not appear in the Census dataset, then they are assigned a race of *Unknown*.

### Final dataset

For those individuals in AA2017 who had duplicate records due to multiple affiliations, we selected one record at random and excluded others. We also removed records that were not assigned a value for their Scientific Age in AA2017 for which no gender could be assigned, and which had fewer than three reviews on *RateMyProfessor.com*. We excluded faculty who had fewer than five reviews in order to mitigate noise. The final matched dataset contained 18,946 records. Finally, we enriched these data with university characteristics from the 2018 Carnegie Classification of Higher Education Institutions. Analysis was conducted on a set of relevant variables extracted from the matched and enriched dataset. Descriptions of these final variables, identified following an extensive literature review of factors relevant to teaching performance, can be found in Table 1. These variables reflect a range of individual, classroom, university, and professional characteristics of the faculty and their teaching. These data, and the code for processing it, can be found at https://github.com/murrayds/aa_rmp.

**Table 1:** Description of final variables. Extracted from *RateMyProfessor.com* (RMP2018), the 2017 version of *Academic Analytics* (AA2017), and the Carnegie Classification of Higher Education Institutions (Carnegie) for matched profiles.

Variable	Source	Description
Overall Quality	RMP2018	The average of all 1-5 point reviews of overall quality left for a professor on *RateMyProfessor.com* between 2012 and 2017. Ratings are aggregated across all courses
Difficulty	RMP2018	The average of all 1-5 point reviews of difficulty left for a professor on *RateMyProfessor.com* between 2012 and 2017. Ratings are aggregated across all courses
Interest	RMP2018	The average of all 1-5 point reviews of student interest left for a professor on *RateMyProfessor.com* between 2012 and 2017. Original levels marked by an order set of five qualitative levels. These levels were mapped to values between 1 and 5 to accommodate numeric calculations. Ratings are aggregated across all courses
Number of reviews	RMP2018	The number of reviews left for the professor between 2012 and 2017. We use this as a control variable
Mentions Accent	RMP2018	True if the word “accent” appears at least once in the text of reviews for an individual
Mentions TA	RMP2018	True if the word “TA” or “Teaching Assistant” appears at least once in the text of reviews for an individual
Has Chili Pepper	RMP2018	True if the individual is given a “chili pepper” symbol, implicitly a rating of physical attractiveness
Gender	Mixed	Gender assigned to each individual of the dataset. Assigned using pronouns included in comments from RMP2018 data
Inferred Race	Mixed	Inferred race assigned to each individual in the dataset based on their family name.
Discretized: Citedness; Output; Awards Won; Grants Held	AA2017	Four variables: Citedness, scholarly output, awards won, and grants held. Each variable represents a count of recent field-normalized research items, categorized into three discrete groups. More detail on how each of these research items is counted by AA is included in supplementary information. Assigned category of “None” if no research item. Assigned “Moderate” if not None, and if between the 1st and 90th percentile (inclusive) of those with at least one of that research item; assigned “High” if greater than 90^th^ percentile
Scientific Age	AA2017	Number of years, in decades, since the individual obtained their terminal degree
Discipline	AA2017	High-level discipline of individual. One of Natural Sciences, Medical Sciences, Social Sciences, Engineering, or Humanities. In case a user was assigned to multiple disciplines, one was randomly selected
Rank	AA2017	The professional rank of the individual, coded as Associate, Assistant, or Full
Uni. Type	Carnegie	The classification of the research activity of the institution: R1 or Not R1
Uni. Control	Carnegie	The classification of the “control” of the institution that the individual is affiliated with: Public or Private

## Results

**Fig 1:**
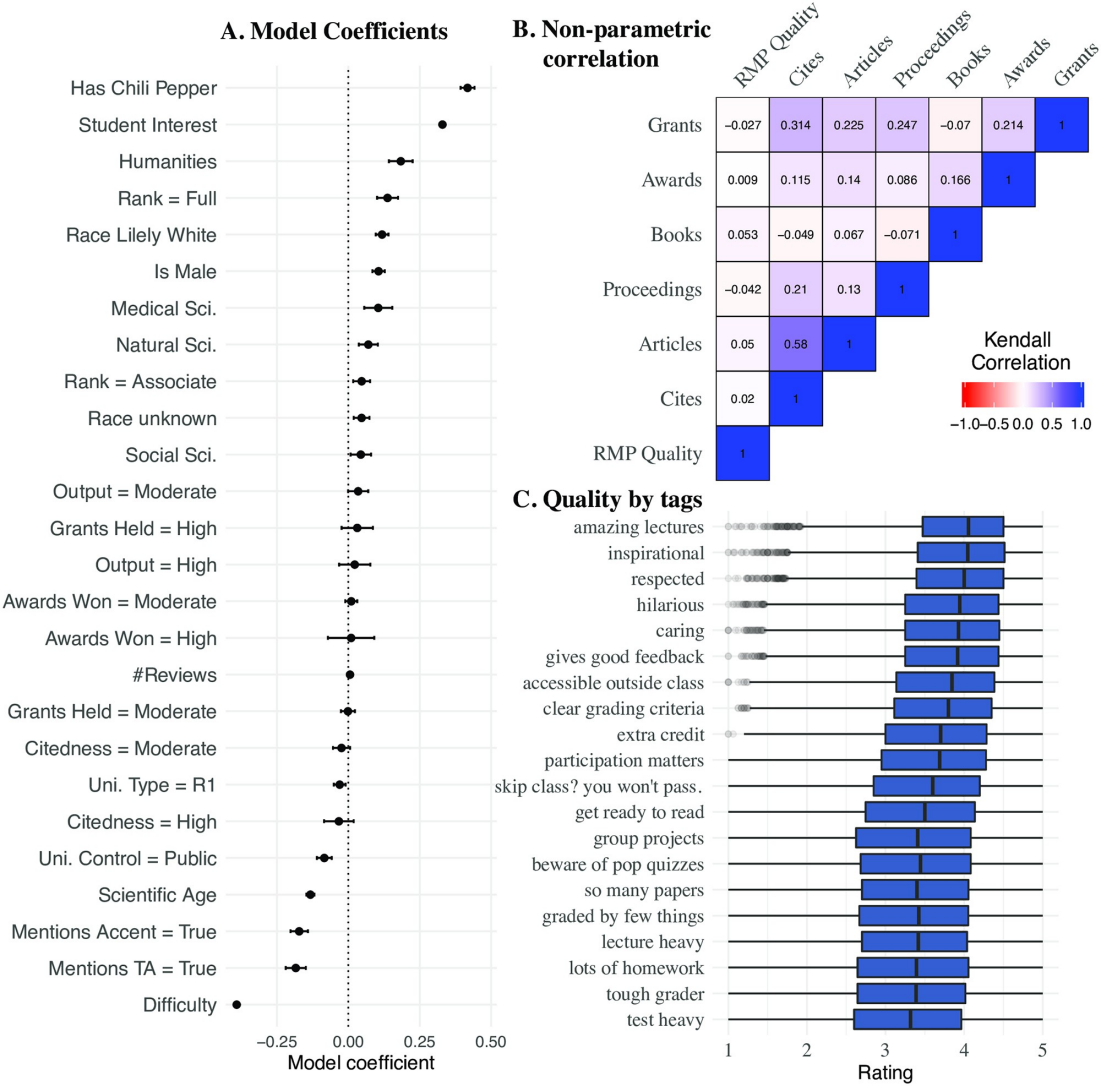
Individual, classroom, university, and research characteristics associated with overall teaching quality. **A**. Estimates of linear regression model using the overall teaching quality (continuous, 1-5) as the response and all variables from Table 1 as the predictor variables. The x-axis corresponds to the estimate for each covariate, which are listed along the y-axis. For binary variables, “false” is always used as the reference level. For Gender, “female” is used as the reference. For race, “Non-White” is used as the reference. For “Rank”, “Assistant” is used as the reference. For Discipline, “Engineering” is set as the reference. For Uni. Control, “Private” is used as the reference. For Uni. Type, “Not R1” is used as the reference. For all research indicators, “Low” is used as the reference. Error bars surrounding each point correspond to the 95^th^ percentile confidence intervals. Results are also shown in S4 Table. **B**. The non-parametric Kendall Rank Tau between research indicators and overall teaching quality. Values map to the correlation between 1 (correlated) and -1 (inversely correlated). Raw values for this test can be found in S5 Table. **C**. The distribution of overall teaching quality ratings for faculty possessing each of the pre-defined “tags” listed on their *RateMyProfessor.com* profile.

We fit a linear regression model with the overall teaching quality as the response, and all other variables from Table 1 as predictors. The resulting model had a *R*^2^ of 0.514. Fig 1A visualizes the estimates of this regression (also shown in S4 Table). Because this is an exploratory analysis, we do not report p-values or significance levels for parameter estimates.

Several personal characteristics of faculty were associated with ratings of overall teaching quality in RMP2018. Presence of the “chili pepper” in RMP2018, which implies attractiveness, was associated with 0.41 point higher overall teaching quality (*β* = 0.42, 95% CI = [0.39, 0.44]); this was the largest positive estimate from the model. Compared to female faculty, male faculty were associated with 0.11 points greater overall teaching quality (*β* = 0.11, 95% CI = [0.08, 0.13]). Faculty having a commonly White surname were associated with 0.118 points greater overall teaching quality (*β* = 0.12, 95% CI = [0.10, 0.14], whereas those with unknown race were associated with slightly higher ratings (*β* = 0.05, 95% CI = [0.019, 0.074]). Faculty who were mentioned as having an accent in a comment left on their RMP2018 profile were associated with 0.17 point lower ratings of overall quality than those for whom an accent was not mentioned (*β* = −0.17, 95% CI = [−0.20, −0.14]). Scientific age was negatively correlated with overall teaching quality such that each additional decade was associated with 0.13 point lower rating (*β* = −0.13, 95% CI = [−0.15, −0.12]). Professional rank had some association with ratings of overall teaching quality. Compared to assistant professors, full professors were associated with 0.14 point higher ratings of overall teaching quality (*β* = 0.14, 95% CI = [0.1, 0.17]); associate professors were associated with only 0.05 point higher ratings (*β* = 0.047, 95% CI = [0.017, 0.076]). Personal characteristics may also interact; for example, we observe evidence that White male faculty are higher than their Non-White, female counterparts (*β* = 0.051, 95% CI = [0.002, 0.10]), among other weaker interaction effects (S6 Table).

Characteristics of the class itself were also associated with ratings of overall teaching quality. The rated difficulty of the course was largest negative estimate from the model; each additional point of difficulty was associated with 0.39 lower points of overall teaching quality (*β* = −0.39, 95% CI = [−0.40, −0.38]). The student interest ratings of a faculty was the second largest positive estimate; each additional point in interest was associated with 0.33 points higher overall teaching quality (*β* = 0.33, 95% CI = [0.32, 0.34]). Faculty for whom a comment on RMP2018 mentioned a teaching assistant were associated with 0.18 point lower ratings of overall quality (*β* = −0.18, 95% CI = [−0.22, −0.15]).

Associations between university characteristics and ratings of overall teaching quality were found to be weaker than for individual and class characteristics. Compared to all others, R1 universities—doctoral universities with very high research activity (as identified by the Carnegie Classification of Higher Education Institutions)—were associated with 0.03 point lower ratings of overall quality (*β* = 0.03, 95% CI = [−0.05, −0.01]. Compared to those in private universities, faculty affiliated with public universities were associated with 0.08 point lower teaching evaluations (*β* = −0.08, 95% CI = [−0.11, −0.06]).

There were notable differences in ratings of overall teaching quality between disciplines. All other disciplines were rated as having higher overall quality than Engineering, the reference level; Engineering was chosen as the reference because it had the lowest ratings of overall teaching quality. Compared to Engineering, faculty in the Humanities were associated with 0.18 point higher overall quality ratings (*β* = 0.18, 95% CI = [0.14, 0.23]). After the Humanities, faculty in Medical Science were associated with 0.11 points higher ratings than those in Engineering (*β* = 0.11, 95% CI = [0.056, 0.153]), followed by faculty in the Natural Sciences (*β* = 0.07, 95% CI = [0.037, 0.10]) and finally faculty in the Social Sciences (*β* = 0.044, 95% CI = [0.008, 0.079]). Distinct disciplinary contexts can also interact with other variables. While a complete cross-disciplinary analysis is out of the scope of the present study, we conduct a preliminary analysis of how gender interacts with discipline. We found that while male faculty get higher ratings, and Humanities and Natural Science faculty are rated more highly than those in Engineering, this total disparity fell when considering the Male/Humanities and Male/Natural Sciences combinations of factors (S6 Table).

Research indicators were only weakly or trivially associated with ratings of overall research quality. During analysis, we designated three levels of field-normalized research productivity over the past 5 years: no publications, moderate (at least one publication, less than or equal to the 90^th^ percentile), and high (above the 90^th^ percentile); this was repeated for all research indicators. Compared to faculty with no publications in the past five years, faculty with moderate publication were associated with 0.034 point higher ratings (*β* = 0.034, 95% CI = [−0.001, 0.069])—this was the only estimate for which confidence intervals only barely crossed zero. Those with a high level of publications were associated with 0.022 point higher ratings (*β* = 0.022, 95% CI = [−0.033, 0.077]). Faculty with a moderate and high level of citations were associated with 0.024 point (*β* = −0.024, 95% CI = [−0.054, 0.006]) and 0.033 point (*β* = −0.033, 95% CI = [−0.085, 0.018]) lower teaching evaluations, respectively. Faculty with a moderate amount of grants were associated with only 0.002 point lower ratings (*β* = −0.002, 95% CI = [−0.026, 0.023]) whereas those with a high amount of grants were associated with 0.031 point higher evaluations than those with no grants (*β* = 0.031, 95% CI = [−0.024, 0.086]). Finally, compared to those with no awards, those with a moderate amount of awards were associated with 0.01 point higher ratings of overall teaching quality (*β* = 0.01, 95% CI = [−0.011, 0.031]), and those with a high amount of awards were associated with a similar 0.01 point higher ratings (*β* = 0.01, 95% CI = [−0.071, 0.091]).

One limitation of this regression analysis was that research indicators, due to their zero-inflated and heavily-skewed distributions, were binned into one of three categorical values; this made them more amenable for analysis but could mask linear relationships. We sought to further assess the presence of the research-teaching nexus by repeating the regression analysis with continuous, rather than categorical variables for research performance indicators (results provided in S7 Table). However, this analysis provided no new evidence for the research-teaching nexus, presenting at most a trivial positive relationship between the field-normalized count of awards and the overall teaching quality (*β* = 0.008, 95% CI = [0.001, 0.014]). We computed an ANOVA test to compare the two approaches but observed no significant difference in the variance explained by the models (p = 0.47). To mitigate the potential impact of multicollinearity, we also performed a regression model using the principal component of the continuous research indicators but still observed no evidence of a relationship between this variable and ratings of overall teaching quality. Additionally, we observed no strong evidence of multicollinearity from the adjusted generalized variance inflation factors of both the model with discrete indicators (S8 Table), and the model with continuous indicators (S9 Table). We also sought to assess the impact of omitted variable bias to see how the absence of research indicator could impact other estimates, but observe only trivial differences, with an ANOVA between the basic model and the model with discrete indicators (Fig 1A) showing no evidence of a difference (*p* = 0.49).

We also investigated the extent to which continuous research performance indicators were correlated with ratings of overall teaching quality using the non-parametric Kendall Rank Tau test (Fig 1B). Non-parametric approaches may be better suited to understanding these zero inflated and skewed data. We calculated the correlations for all combinations of research indicators and separated total publication count into three variables corresponding to the count of articles, count of conference proceedings, and count of books indexed in AA2017 (these variables are described in S1 Table). However, we observed only trivial correlations between research indicators and ratings of overall teaching quality, the strongest having a value of 0.046 for the number of articles, followed by the number of books. For faculty with positive research indicators, we investigated the distribution of overall teaching quality by decile rank (S2 Fig) which revealed some evidence of a positive linear relationship between overall teaching quality and citations and publications. However, as these results did not bear out when partitioning by discipline (S3 Fig), when the linear trends all but disappeared; these small correlations may be confounded by disciplinary differences in publishing patterns and teaching quality. We note that the research indicators collected by *Academic Analytics* include only recent performance (5 years for publications and citations, 10 years for books) and do not represent faculty’s full career, which may have proven more predictive of ratings of teaching quality.

Having observed the large estimates of individual and class characteristics from our regression analysis, we further investigated which characteristics of teaching were associated with the *RateMyProfessor.com* overall teaching quality rating. The website allows allows users posting a review to select from a 20 pre-defined tags that denote common characteristics of university faculty and classes. Fig 1C shows the distribution of overall quality scores for faculty having each of these tags. The tags associated with the highest ratings of overall teaching quality tend be personal characteristics of the instructor such as “amazing lectures”, “inspirational”, “respected”, “hilarious”, and “caring”. The tags associated with the lowest ratings instead tend to refer to course characteristics, such as “graded by a few things”, “lecture heavy”, “lots of homework”, “tough grader”, and “lots of tests”. The results from these tags confirm the relationship between difficulty and ratings observed in the regression model.

## Discussion

Ideally, faculty evaluation would be an unbiased performance assessment, uninfluenced by gender, ethnicity, age, attractiveness, or other personal characteristics. However, empirical analyses of student evaluations of teaching (SETs) have demonstrated that they often fall short of this ideal [3–12]. Moreover as the ideal of the university posits a mutually beneficial research-teaching nexus, faculty evaluation should be holistic, considering performance across all professional responsibilities; however, assessments of the so-called research-teaching nexus have not produced a clear consensus of its presence, nature, or extent [28–34]. By constructing a large and heterogeneous dataset of tenure and tenure track faculty in the U.S., this exploratory study provides additional evidence of bias in SETs while also demonstrating little to no relationship between common indicators of teaching and research.

### Individual characteristics

The strongest correlate with teaching evaluations was whether or not the faculty had a “chili pepper” rating on *RateMyProfessor.com*. The precise implication of the chili pepper is unclear, as it was never explicitly defined and so its meaning will vary between users. We conceive the “chili pepper” as a rating of the physical attractiveness of the instructor, following past research [46] and widespread cultural understandings [21]. Following controversy, this rating was removed in 2018 (see S1 Text) however it remained in use at the time of data collection. Our finding is consistent with studies of student evaluations in traditional evaluative settings [7, 47], studies of faculty’s online self-presentation [48], and past studies of *RateMyProfessor.com* [10, 46]. In unbiased evaluation, a faculty’s physical attractiveness should not factor into the quality of their teaching or pedagogical skill. The relationship we observed could result from student’s implicit bias favoring physically attractive faculty. It can also be interpreted as a “halo effect” [49], whereby student’s positive impressions of one aspect of their professor (e.g.: their attractiveness) influences other aspects of their evaluation. Student’s perceptions of physical attractiveness are also likely to differ with the perceived age, race, and gender of both the instructor and the students [50], resulting in different manifestations of this trend across different contexts. For example, younger faculty were more likely to be assigned a chili pepper, demonstrated by the negative trend between scientific age and probability of having a chili apparent in S4 Fig). While we control for some of these characteristics (e.g., age, gender), we cannot effectively control for others such as ethnicity and student demographics.

We observed a small trend such that male faculty tended to receive higher ratings (of 0.10 points) of overall teaching quality than female faculty. Past studies of traditional SETs have noted gender biases favoring men in experimental settings [8] and in large-scale observational studies [5]. Studies leveraging *RateMyProfessor.com* have observed gendered differences in language used to describe faculty [51] but findings of bias in evaluation scores have been mixed with reports of small or no significant gender bias depending on context [30, 46]. We observed no evidence of gender difference in the distribution of overall ratings based on aggregate data (S5 Fig), but did observe a relationship when controlling for other variables such as scientific age, disciplines, and university context (Fig 1A); This discrepancy and the lack of consensus among studies suggests that gender bias in SETs is contingent on contextual factors of the university, discipline, and student body [3].

Faculty with commonly-White family names tended to be rated more highly than others. This finding affirms past studies that identified racial bias in SETs such that persons of color, particularly black faculty, were rated lower than their White counterparts [6, 9, 52]. However, those with names absent from the U.S. Census data also tended to have higher ratings than Non-White faculty; we cannot speak to the precise demographics of these names, however these names were more common in fields such as Engineering (S6 Fig), which also tended to have the most Non-White associated family names (S7 Fig). It is likely that the “unknown” category is therefore a mixture of White and Non-White faculty, the precise demographics of which require further investigation. We found evidence that race and gender interact, such that White Male faculty tended to be rated more highly than others, mirroring inter-sectional narratives. Related to race, faculty for whom an “accent” was mentioned in their evaluations tended to be rated lower than those for whom no accent was mentioned. *RateMyProfessor.com* and *Academic Analysis* offer no means of reliably inferring country of origin of faculty; here, we consider the mention of an accent as a proxy indicating non-native English speaker who may encounter bias and stereotyping in SETs. Whereas students often claim that instructor’s accent is less important than their knowledge of the source material [53], accented faculty have been found receive lower evaluations, especially for comprehension [54]. On *RateMyProfessor.com*, a population of Asian-born professors (who may or may not have noticeable accents) were found to receive lower ratings than their U.S. born counterparts [55]. Non-White and foreign-born faculty face additional challenges when teaching such as stereotyping and prejudice. These approaches, while limited, demonstrate how biases can manifest in student’s evaluations of faculty, which can hinder their career and produce additional inequality.

More senior faculty, in terms of the number of years since obtaining their Ph.D., tended to receive lower ratings; each additional decade of scientific age was associated with 0.13 point lower score. Most past research studying the relationship between age and SETs has studied actual age, a value which is likely correlated with the scientific age we study here. One study of data from *RateMyProfessor.com* found evidence that older instructors were rated lower, but that this effect disappears after controlling for other factors, such as their physical appearance and the difficulty of their courses [11]. However, even after controlling for many of the same factors, our findings contribute to the consensus of studies finding that older faculty receive lower evaluations [7, 10].

Related to scientific age is also professional rank; we observed that full professors tended to get higher ratings than both assistant and associate professors, contrary to what we would expect given that younger faculty receive higher ratings. Assistant professors tended to be scientifically younger, whereas full professors tended to be older (shown in S8 Fig). This suggests the relationship between seniority and SET ratings are not necessarily linear, and that those faculty with experience, though perhaps not too much seniority, tend to do best. One past study compared teaching from non-tenured instructors and tenure/tenure track faculty found that non-tenured instructors had stronger evaluations [56]. However, there is little research examining SETs across tenure ranks (assistant, associate, full). Common wisdom suggests that teaching benefits from experience but evidence suggests that past a baseline level of experience, students generally rate younger professors more highly over more senior and experienced faculty. However, younger professors may more readily relate to students or employ more recent pedagogical techniques. Moreover, the requirements, demands, and roles of faculty change over the course of their career, and teaching may be de-emphasized during certain career stages.

### Classroom characteristics

The strongest negative relationship we observed was between overall teaching quality and ratings of class difficulty. Every point increase in difficulty rating (where five is most difficult, and one is easiest) was associated with a drop of nearly half a point in overall quality. This finding is consistent with past studies identifying a negative relationship between difficulty and quality ratings in traditional SETs [57] and on *RateMyProfessor.com* [46, 57, 58]. One interpretation of this finding is that *RateMyProfessor.com* is a site used by students to complain about difficult courses and low grades, but overall teaching quality scores are actually somewhat skewed towards higher ratings, with median ratings of 3.6 for the matched dataset. Others have suggested that students have varying definitions of “difficulty”. For example, in some studies of SETs, difficulty was associated with perceptions of “fairness” in the course [57, 59]; similar effects were observed on *RateMyProfessor.com* [37]. Other scholars have found that clarity of course material and expectations are also important factors of student’s ratings of difficulty when posting reviews [36, 58]. The form for posting a review on *RateMyProfessor.com* is vague, and so there are boundless interpretations of the difficulty scale, which we cannot directly examine. However, tags associated with low teaching quality (Fig 1C) tended to relate to quantity and type of course material and grading (“tough grader”, “lecture heavy”, “lots of homework”, “test heavy”).

Ratings of prior interest almost mirrored those of difficulty, and were the second largest positive correlate with overall teaching ratings; each additional point in student interest was associated with 0.36 point higher ratings. Past studies found similar results when investigating SETs [60] and *RateMyProfessor.com* [46], though generally little research has been conducted examining the effect of student’s prior interest. Under the U.S. liberal arts model of higher education, many instructors will teach courses containing a mixture of students with radically different interest levels in the curriculum, from majors in the subject field to students fulfilling general education credits. This dynamic may similarly affect SETs. Indeed, there is some, if limited, evidence that elective courses (which are freely chosen by the student) often receive better student ratings than required courses [61]. Faculty who teach required or general-education courses may be at a systematic disadvantage during performance evaluations if they are subject to the prior interests of their students. However, there are also difficulties with interpreting the rating of “prior interest” because it assumes that the student is aware of their true interest in a course at the time of posting their review, and that this measure is somehow indicative of their intrinsic interest in the subject. As with the “chili pepper”, ratings of interest may instead reflect a halo effect, such that a student’s rating of “interest” (or other teaching-related categories) is more closely related to their opinion of the professor than the course material.

We observed that faculty whose reviews mentioned a teaching assistant (TA) received lower ratings than those where no TA was mentioned. The presence of a TA is our best (though highly flawed) proxy for whether an instructor teaches large courses as TAs are typically employed for larger classes (though not in all cases, and with variations by discipline and university context). Our finding is however consistent with past studies that observed a small but significant negative effect between class size and SET ratings [4, 62, 63]. However, it is difficult to disentangle the extent that the TA in *RateMyProfessor.com* reviews indicates of the course size, or whether students only mentioned TAs when they were a negative aspect of the course.

### University characteristics

Affiliation with public universities was related with lower ratings than affiliation with private universities, by about 0.08 points of overall quality. One reason for this small difference might be that faculty at private universities have been found to give, on average, higher grades to their students [64] and this higher expected grade may positively influence subsequent evaluations [65]. However, the difference we observed might also emerge from the distribution of contextual factors across public and private universities. For example, the sample of private colleges may include many smaller or liberal-arts colleges hosting more faculty in the Humanities and Social Sciences.

We also examined the research classification of universities, but we observed only trivial differences between R1 and non-R1 universities. There is little research examining differences in SETs across different university types, whether between public and private universities or between research-focused and teaching-focused. Part of this may be because aggregating SETs across institutions is difficult due to their sensitivity. The formats of SETs are also likely to vary between institutions making comparisons between universities difficult. Here we find little difference in ratings of teaching quality based on university types, but more work is needed to understand the role of institutional context in teaching.

### Discipline

We observed distinct trends in student’s ratings of teaching by discipline; faculty in the Humanities tended to be the highest rated, whereas faculty in Engineering and Social Science tended to have the lowest ratings. These findings are consistent with past studies of discipline and teaching evaluation. For example, faculty teaching traditionally quantitative disciplines were found to receive lower ratings, an effect that was observed for traditional evaluations [13, 17] and on *RateMyProfessor.com* [46]. However, whereas Social Science is not typically associated with quantitative courses, we observed that teaching ratings for faculty in Social Science tended to only be trivially higher than faculty in Engineering. One reason for this discrepancy may be that the high-level classifications used in this study mask the true heterogeneity of disciplines and courses and don’t easily allow for “quantitative” / “not quantitative” distinctions. However, we also observed that regression estimates for disciplinary effects differed from the simple average of ratings by discipline, for which Natural Sciences actually have the lowest median rating (S5 Fig); this suggests that some of some of the disciplinary differences might be explained by contextual factors such as the distribution of faculty demographics, classroom, and university characteristics across disciplines. For example, in our preliminary analysis of the interaction between gender and discipline, we observe differences across fields. More thorough work is necessary to understand discipline and course topic relates to teaching and SETs; in particular, a more comprehensive and thorough statistical analysis according to discipline, combined with a more fine-grained disciplinary classification could provide additional insight into the relationship between discipline and SETs.

### Research-teaching nexus

Applying several different techniques, we observed little to no relationship between indicators of research performance and ratings of overall teaching quality on *RateMyProfessor.com*. In other words, we observed evidence consistent with a neutral research-teaching nexus, as observed in several past studies [34, 66–69]. In the study most similar to our own, a weak correlation was observed between ratings on *RateMyProfessor.com* and journal publication count [30], however the study examined only faculty affiliated within Marketing departments. Other studies have observed positive research-teaching nexus between SETs and research productivity under certain circumstances [70, 71], but generally, empirical evidence is lacking [34]. The results from our analysis contribute to the consensus of a neutral relationship between research and teaching.

The research-teaching nexus is complicated, and difficult to assess. Evidence for a null model tend to use SETs or an equivalent indicator to measure teaching performance. Studies also tend to use output-based bibliometric indicators to measure research performance; our study also only examines recent research output, whereas longer time-scales of output may correlate more strongly with teaching. Such indicators have been called into question as being improper or inadequate tools that don’t measure true teaching or research performance [42, 72, 73].

The research-teaching nexus, if it exists, may be intangible or may not manifest in performance measures. Rather than further attempting to empirically verify the existence of the research-teaching nexus using quantitative tools, qualitative methodology may prove more useful to explore perceptions of the nexus [24–27, 74]. Such approaches could reveal the extent to which faculty believe the nexus exists, what they believe about the nature of the nexus, and how the nexus has evolved with increasing faculty time constraints [31, 32, 75]. Moreover, if the relationship between research and teaching is held as a value of academia, then researchers and administrators should explore ways of actively promoting the research-teaching nexus [66].

### Limitations

Our study is subject to several limitations. First, we note that we conducted a preliminary and exploratory study using observational data and as such our methods were not pre-registered and our analysis is subject to issues of multiple comparisons.

Second, our use of *RateMyProfessor.com* as a proxy for SETs is a clear limitation, as reviews on the website suffer from issues of external validity [58] and selection bias, wherein students with extreme opinions are likely to be the ones to post reviews. The website has also endured criticism that reviews not align with effective teaching [36]. While traditional SETs are intended for internal use for faculty evaluation and improvement, the primary purpose of *RateMyProfessor.com* is to help students select courses; the expectations and rating criteria of each likely diverge. Despite these issues, ratings on *RateMyProfessor.com* have been found to correlate with traditional SETs (see S1 Text). Similarly, quantitative measures of teaching and research do not capture quality. The indicators used in this analysis are also limited in that they capture only recent performance—an artifact of *Academic Analytics*—more insights may be gained by examining the historical trends of professor’s research or teaching performance.

Third, we were limited by the evolving nature of our data sources. *RateMyProfessor.com* has undergone many changes since its inception, including changes to the features and indicators provided to raters. While we limit our analysis to relatively recent reviews, during this time certain indicators (such as the separate measures of “Clarity” and “Helpfulness” and “Interest”, as well as the “Chili Pepper”) were removed (see S1 Text).

Fourth, by limiting our analysis to tenure and tenure-track faculty in the United States, our analysis excluded contingent and other non-tenure track faculty who comprise over 70 percent of the U.S. [76] and more than 50 percent [77] of Canadian faculty appointments, as well as graduate student instructors who may teach a large proportion of courses [78]. These populations face unique challenges [79, 80] that remain unaddressed in the present study. Moreover, these results are limited to faculty within the United States, and so our findings may not generalize to other national contexts.

Finally, our analysis was also limited by the record-matching algorithm which did not capture all relevant faculty. The parameters for record matching favored precision over recall, so the number of matched faculty are a conservative sampling of the population. Additionally, there were many professors who simply did not appear on *RateMyProfessor.com* or in *Academic Analytics*, and so do not appear in the present analysis. Given that there is no known list of all U.S. faculty, it is difficult to assess the extent to which the matched faculty were representative of U.S. tenure and tenure-track faculty as a whole.

## Conclusion

This paper provided an exploratory analysis of the factors relating to online ratings of teaching quality and their relationship to research productivity. We constructed a novel dataset by matching records of known tenure and tenure-track faculty from *Academic Analytics* with individuals listed on *RateMyProfessor.com*. We assessed the effect of the demographics of the teacher, characteristics of the class, of the university, and of the discipline. Faculty tended to receive higher ratings when they were rated as attractive (having the “chili pepper” on *RateMyProfessor.com*), when they were male, when they were young, when they were not mentioned as having an accent, and when they were full and associate professors. Faculty tended to receive lower ratings when the course was difficult, when there was little student interest, or when a teaching assistant was mentioned. We observed some evidence that faculty in private universities were rated slightly higher than those from public universities, but overall university characteristics were weakly related to ratings of teaching. Faculty from the Humanities tended to be rated most highly, followed by those in the Medical Sciences, Natural Sciences, Social Sciences, and finally Engineering.

In addition to demographic and contextual factors, we also assessed the presence and extent of the so-called *research-teaching nexus*, the relationship between research and teaching. Comparing indicators of recent publications, recent citations, current grant funding, and professional awards, we found evidence consistent with a *neutral* nexus, or no relationship between research and teaching.

These results and data provide a foundation for future large-scale analysis of SETs and of the research-teaching nexus. Future work could delve deeper into this data, comparing patterns of student ratings of teaching across more disciplines, university types, course levels, and even specific departments. *RateMyProfessor.com* also offers a trove of text data from student comments; content analysis and text mining of these data could reveal key insights to the underlying factors of student’s ratings, such as gendered language and attitudes [51]. This text data can be leveraged to identify other faculty characteristics, such as their self-disclosed or perceived LGBTQ+ status, allowing study into the unique challenges faced by those faculty of different sexual orientations and gender identities [81–83]. The current dataset could also be enriched with survey data relating to time spent on service-related activities or more detailed bibliometric indicators from the Web of Science or Scopus. It is our hope that the present analysis is the first of many to explore broad trends in the nature of quantitative performance measures across disciplinary, university, and classroom contexts.

Despite controversy, student evaluations of teaching dominate faculty evaluation across the United States; given their continued importance, it is important to understand what factors contribute to these scores and how these factors differ between institutional and disciplinary contexts. Our results build on past research that demonstrates the biases, limitations, and deficiencies of SETs. The confluence of research should cause the higher education community to consider whether the student evaluations of teaching should be discounted, rehabilitated, or done away with all together.

## Supporting information

S1 Text (PDF)

S1 Fig Distribution of research indicators. A point-based histogram of frequencies of research indicator values in the dataset placed on a LogLog scale. Each point plots the frequency of professors with a given “count” of research items. Non-normalized raw counts are used. Points are grouped by discipline, specified by color and shape. Aggregate values by discipline can be found in S11 Table.
(TIF)

S2 Fig Ratings of teaching quality by research performance. Boxplots of ratings of overall teaching quality for faculty having a positive non-zero value for field-normalized research indicators. Indicator performance is binned into deciles (x-axis). The horizontal grey line is the median for faculty with a value of zero in each indicator. The red line corresponds to the median rating of overall teaching quality for faculty in each decile bin.
(TIF)

S3 Fig Ratings of teaching quality by research performance and discipline. Boxplots of ratings of overall teaching quality for faculty having a positive non-zero value for field-normalized research indicators. Indicator performance is binned into deciles (x-axis), repeated for faculty in each of the five discipline categories. The red line corresponds to the median rating of overall teaching quality for faculty in each decile bin.
(TIF)

S4 Fig Younger faculty more often assigned chili pepper. The proportion of faculty in the matched dataset that were assigned a chili pepper (y-axis), implicitly suggesting attractiveness, by scientific age (x-axis).
(TIF)

S5 Fig Distribution of teaching quality across categorical variables. The distribution of ratings of overall teaching quality (y-axis) for values of each categorical variable (x-axis) from the matched dataset. Includes discipline, gender, whether the faculty has a chili pepper, inferred race, the university type, the university control, and the professor’s rank.
(TIF)

S6 Fig Proportion of faculty with unknown race by discipline. (TIF)

S7 Fig Faculty demographics by discipline. (TIF)

S8 Fig Distribution of faculty scientific age by rank. Boxplots for the distribution of scientific age (years since earning PhD or other terminal degree) and the rank of faculty, as indexed in AA2017.
(TIF)

S9 Fig Matched rated more poorly, more difficult, with more comments. Boxplots detailing the distribution of overall quality, difficulty, interest, and the number of comments for individuals from RMP2018 were unmatched (white) vs. matched to records in AA2017 (dark grey). Labels in each boxplot state the median.
(TIF)

S1 Table Description of relevant variables from academic analytics 2016 dataset. (PDF)

S2 Table Description of relevant variables extracted from RateMyProfessor.com reviews. (PDF)

S3 Table Description of relevant variables extracted from RateMyProfessor.com teacher profiles. (PDF)

S4 Table Results of multiple linear regression model. (PDF)

S5 Table Results of Kendall Rank Tau. (PDF)

S6 Table Results of multiple linear regression model with interactions. (PDF)

S7 Table Results of multiple linear regression model using continuous research performance indicators. (PDF)

S8 Table Little evidence of multicollinearity in discrete regression model. (PDF)

S9 Table Little evidence of multicollinearity in continuous regression model. (PDF)

S10 Table Little difference between population of matched and unmatched academic analytics faculty. (PDF)

S11 Table Average counts of research item, by discipline. (PDF)
