# Supplementary material for: Exploring the personal and professional factors associated with student evaluations of tenure-track faculty
Source: PLoS One. 2020 Jun 3;15(6):e0233515. doi: 10.1371/journal.pone.0233515 (PMC7269236; doi:10.1371/journal.pone.0233515)
Supplement: S1 Text — (PDF) [file pone.0233515.s001.pdf]

S1 Text

Values no longer used by RateMyProfessor.com.

Before summer, 2017, when a user left a review for a professor on *RateMyProfessor.com*, they were prompted to select their interest level prior to attending class. The available interest levels were grouped into five ordinal categories, ranging from “Low” at the bottom to “It’s My Life” at the top (further description available in S2 Table and S3 Table. The selected interest level was then displayed on each user’s review. Since the summer of 2017, this feature is no longer present on the site. We however have data on student interest for every review beforehand, and so we do not exclude this variable form our analysis.

Clarity and helpfulness were two other quantitative metrics in use by *RateMyProfessor.com*, but were recently removed from the site. Though we have these data, we exclude them from our present analysis because past research, and our own analysis, demonstrates that they are strongly correlated with overall quality [1].

The ”chili pepper” or ”hotness” rating, while never explicitly defined as such, was implicitly associated with the physical attractiveness of the professor. This rating was removed from *RateMyProfessor.com* as of 2018, however, it was present throughout the period of data collection and so we include it in our analysis.

Validity of Academic Analytics and RateMyProfessor.com.

As *Academic Analytics* is used by more institutions, its validity has been called into question [2]. However, these allegations have largely been anecdotal given that the data is proprietary and thus not available for public scrutiny. A large-scale validation exercise remains necessary to thoroughly assess the accuracy of research indicators in AA2017. One such analysis has already been conducted on CrossRef—the source of AA2017’s publication and citation network [3]; this analysis found considerable overlap of both citations and publications with more widely accepted research evaluation databases such as Scopus and Web of Science. Our own small-scale analysis, compared the counts of items listed on the CVs of professors to their counts listed in AA2017 and demonstrated reasonably accurate coverage of publications. In light of this evidence, we believe that AA2017, while not thoroughly vetted, is sufficient for large-scale analysis. An advantage of using AA2017 is that they collect data for faculty in a variety of disciplines, potentially leading to greater coverage of Humanities and Social Sciences than traditional bibliometric sources such as Scopus and Web of Science [4].

Concerns have been raised over the validity of *RateMyProfessor.com.* The website lacks external validity as a result of its open and anonymous nature, allowing students to rate a course on their first day of attendance, or even years after [5]. Additionally, the content on the site is entirely user-generated with little to no gatekeeping to ensure that real students are in fact reviewing real professors for courses they actually took. One result of this is entirely fabricated records; for example, *RateMyProfessor.com* included a profile for “Albus Dumbledore, professor of transfiguration at Hogwarts School of Witchcraft and Wizardry”, a popular fictitious character from the *Harry Potter* franchise, who had 143 student ratings at the time of writing. By merging records with *Academic Analytics*, which contains a known list of active tenure and tenure-track faculty, we mitigate the impact of fabricated or otherwise misleading

profiles. Despite criticism, evidence supports that *RateMyProfessor.com* ratings correlate with traditional student-evaluations of teachers [1, 6–9], suggesting that findings from our analysis are likely to generalize to other faculty evaluations.

Representativeness of matched vs. unmatched records in Academic Analytics.

S10 Table details descriptive information for individuals from the *Academic Analytics* dataset (AA2017) who were matched versus not matched with records in *RateMyProfessor.com*. The unmatched statistics includes only tenure and tenure track faculty. This comparison allows us to assess the extent of bias in our matching process.

The largest differences we observed between the matched and unmatched dataset relate to the discipline of faculty and the control of the university (public vs. private), and the rank of the professor. Under the assumption that all students in a course are equally likely to leave their instructor on *RateMyProfessor.com*, professors who teach more classes and are exposed more students would be more likely to appear in RMP2018. Given this, one possible contributing factor to differences between matched and unmatched data is the relative exposure of these faculty; this exposure will likely differ across disciplinary and university contexts. For example, medical scientists were underrepresented in the matched data; a cursory investigation of CVs from these faculty revealed that while they often held affiliations with university medical schools, they focus on research and teach comparatively little. Similarly, public universities tend to be much larger, on average, than private universities, and so faculty are likely to teach larger classes and be exposed to more students, leading to an over-representation of public schools. Even within a university, associate faculty may have greater teaching loads than full faculty, and so would be more likely, on average, to have a review on *RateMyProfessor.com*, resulting in an over-representation of associate faculty in the matched data. Moreover, since S10 Table includes only tenure and tenure-track faculty, departments that use lecturers to teach large courses may be underrepresented; our analysis only concerns tenure and tenure-track faculty and so while lecturers may have many students, they are excluded from analysis.

We observed only small differences in research indicators between matched and unmatched faculty; research performance may differ by faculty's institutional affiliation, seniority, and discipline; we partially control for this last factor through a simple field-normalizing, but this form of normalization is flawed [10]. Minor differences that we observed in indicators of research performance between and unmatched faculty may have resulted from the differences in the distribution of these faculty across university, disciplinary, and professional contexts.

Representativeness of matched vs. unmatched records in RateMyProfessor.com.

Past analyses of faculty rating data from *RateMyProfessor.com* have typically followed one of two approaches: in the first approach researchers examine large samples of profiles sampled from the website [11, 15, 16]; however, results from this approach may be confounded by fake profiles and by mixing profiles of full-time research faculty with those of graduate instructors, lecturers, or part-time faculty. Other studies have instead examined smaller known population, typically limited to faculty from a small number of departments and universities whose profiles can be manually extracted from *RateMyProfessor.com* [1, 5, 8, 17, 18]; however, these studies may lack external validity. The present study attempted a balance between these approaches by examining a large and diverse set of known tenure and tenure-track faculty.

S9 Fig shows how ratings from RMP2018 differ between the population of matched and unmatched faculty. Matched faculty tended to have slightly lower ratings of overall quality (median = 3.7, mean = 3.5) than unmatched faculty (median = 4.0, mean = 3.8). Matched faculty tended to be rated as more difficult (median = 3.2, mean = 3.2) than unmatched faculty (median = 2.8, mean = 2.9). Ratings of student interest were roughly the same between matched and unmatched faculty. The distribution of the number of comments was highly skewed, though matched faculty tended to have more comments (median = 8, mean = 10.2) than unmatched faculty (median = 7, mean = 9.5). However, these values include only individuals who had 25 or fewer reviews, as was used in the main analysis. The distribution of reviews tended to be highly positively skewed, with a maximum value of 268 for matched faculty, and 2,365 for unmatched faculty.

The largest difference between matched and unmatched faculty in RMP2018 was that a larger proportion of unmatched faculty had a chili pepper (30.5 percent with) than matched faculty (19.9 percent with). Assignment of the chili pepper, indicating the attractiveness, is associated with scientific age (see S4 Fig); one possible reason for this difference may be that matched faculty are older than unmatched faculty, however since RMP2018 does not record age this cannot be assessed. Matched faculty more often were mentioned as having an accent (8.7 percent) than unmatched faculty (5.4 percent) though this difference was relatively small. Matched faculty were also more likely to have had a teaching assistant mentioned (5.3 percent) compared to unmatched faculty (1.1 percent).

Differences between matched and unmatched faculty likely resulted from differences in university, disciplinary, and professional contexts that shape who is most likely to be reviewed. Since we matched RMP profiles with records from AA, matched faculty were all tenure and tenure-track faculty at research-oriented universities. The unmatched RMP profiles however contained many faculty from small liberal arts colleges, community colleges, and other teaching-oriented institutions; these institutions may have had different faculty demographics than larger research-oriented institutions. Related to institutional context is discipline—for example, larger research-oriented universities may have been able to host more faculty and students in disciplines requiring lab space or special facilities (e.g.: medicine) or disciplines requiring accreditation (e.g.: civil engineering). Some of these disciplines will be more likely to use teaching assistants. There are differences in faculty demographics between disciplines, based on gender and nationality [12–14] which might relate to mentions of accent. Unmatched faculty consisted of many non-tenure faculty and so would include part-time faculty, non-research instructors, and graduate instructors. These various teacher roles may be associated with distinct demographics and teaching contexts, which may have contributed to the observed differences between matched and unmatched faculty.

References

1. Silva KM, Silva FJ, Quinn MA, Draper JN, Cover KR, Munoff AA. Rate My Professor: Online Evaluations of Psychology Instructors. Teaching of Psychology. 2008;35(2):71–80. doi:10.1080/00986280801978434.

2. Flaherty C. Rutgers Graduate School faculty takes a stand against Academic Analytics; 2016. Available from: `https://www.insidehighered.com/news/2016/05/11/rutgers-graduate-school-faculty-takes-stand-against-academic-analytics`.

3. van Eck NJ, Waltman L, Larivière V, Sugimoto CR. Crossref as a new source of citation data: A comparison with Web of Science and Scopus; 2018. Available

from: https://www.cwts.nl:443/blog?article=n-r2s234&title=crossref-as-a-new-source-of-citation-data-a-comparison-with-web-of-scienc[illegible]

4. Mongeon P, Paul-Hus A. The Journal Coverage of Web of Science and Scopus: a Comparative Analysis. Scientometrics. 2016;106(1):213–228. doi:10.1007/s11192-015-1765-5.

5. Davison E, Price J. How do we rate? An evaluation of online student evaluations. Assessment & Evaluation in Higher Education. 2009;34(1):51–65. doi:10.1080/02602930801895695.

6. Gregory KM. How Undergraduates Perceive Their Professors: A Corpus Analysis of Rate My Professor. Journal of Educational Technology Systems. 2011;40(2):169–193. doi:10.2190/ET.40.2.g.

7. Kindred J, Mohammed SN. ”He Will Crush You Like an Academic Ninja!” Exploring Teacher Ratings on Ratemyprofessors.com. Journal of Computer-Mediated Communication. 2005;10(3):00–00. doi:10.1111/j.1083-6101.2005.tb00257.x.

8. Coladarci T, Kornfield I. RateMyProfessors. com versus formal in-class student evaluations of teaching. Practical Assessment & Research Evaluation. 2007;12(6).

9. Sonntag ME, Bassett JF, Snyder T. An Empirical Test of the Validity of Student Evaluations of Teaching Made on RateMyProfessors.com. Assessment & Evaluation in Higher Education. 2009;34(5):499–504.

10. Ioannidis JPA, Boyack K, Wouters PF. Citation Metrics: A Primer on How (Not) to Normalize. PLoS Biology. 2016;14(9). doi:10.1371/journal.pbio.1002542.

11. Clayson DE. What does ratemyprofessors.com actually rate? Assessment & Evaluation in Higher Education. 2014;39(6):678–698. doi:10.1080/02602938.2013.861384.

12. Larivière V, Ni C, Gingras Y, Cronin B, Sugimoto CR. Bibliometrics: Global gender disparities in science. Nature News. 2013;504(7479):211. doi:10.1038/504211a.

13. Boyle PJ, Smith LK, Cooper NJ, Williams KS, O'Connor H. Gender balance: Women are funded more fairly in social science. Nature News. 2015;525(7568):181. doi:10.1038/525181a.

14. Leslie SJ, Cimpian A, Meyer M, Freeland E. Expectations of brilliance underlie gender distributions across academic disciplines. Science (New York, NY). 2015;347(6219):262–265. doi:10.1126/science.1261375.

15. Rosen AS. Correlations, trends and potential biases among publicly accessible web-based student evaluations of teaching: a large-scale study of RateMyProfessors.com data. Assessment & Evaluation in Higher Education. 2018;43(1):31–44. doi:10.1080/02602938.2016.1276155.

16. Stonebraker RJ, Stone GS. Too Old to Teach? The Effect of Age on College and University Professors. Res High Educ. 2015;56(8):793–812. doi:10.1007/s11162-015-9374-y.

17. Reid LD. The role of perceived race and gender in the evaluation of college teaching on RateMyProfessors.Com. Journal of Diversity in Higher Education. 2010;3(3):137–152. doi:10.1037/a0019865.

18. Carter RE. Faculty Scholarship Has a Profound Positive Association With Student Evaluations of Teaching—Except When It Doesn't. Journal of Marketing Education. 2016;38(1):18–36. doi:10.1177/0273475315604671.
