# Supplementary figures and images for: Exploring the personal and professional factors associated with student evaluations of tenure-track faculty
Source: PLoS One. 2020 Jun 3;15(6):e0233515. doi: 10.1371/journal.pone.0233515 (PMC7269236; doi:10.1371/journal.pone.0233515)

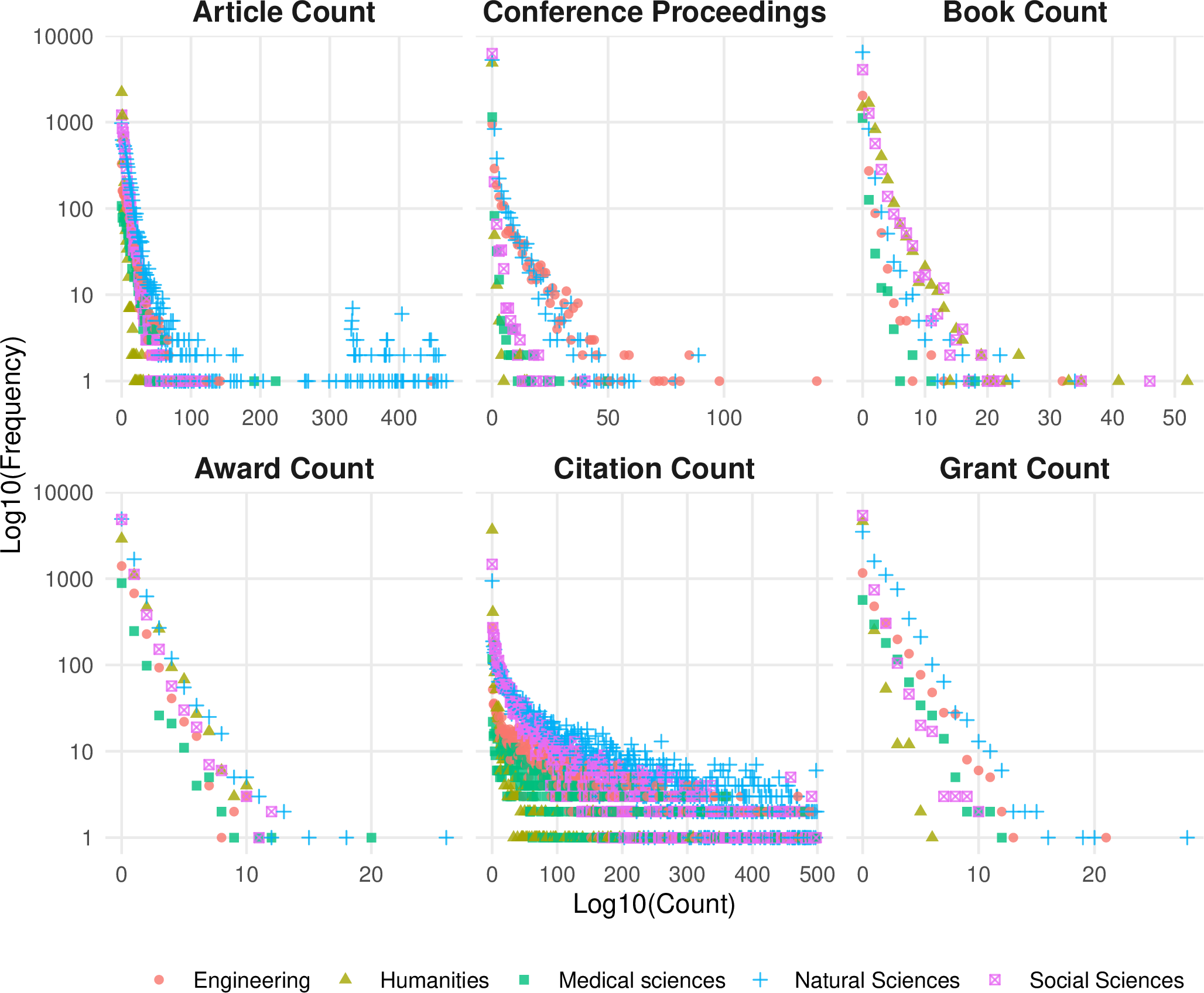

Supplement: S1 Fig — A point-based histogram of frequencies of research indicator values in the dataset placed on a LogLog scale. Each point plots the frequency of professors with a given “count” of research items. Non-normalized raw counts are used. Points are grouped by discipline, specified by color and shape. Aggregate values by discipline can be found in S11 Table. (TIF) [file pone.0233515.s002.tif]

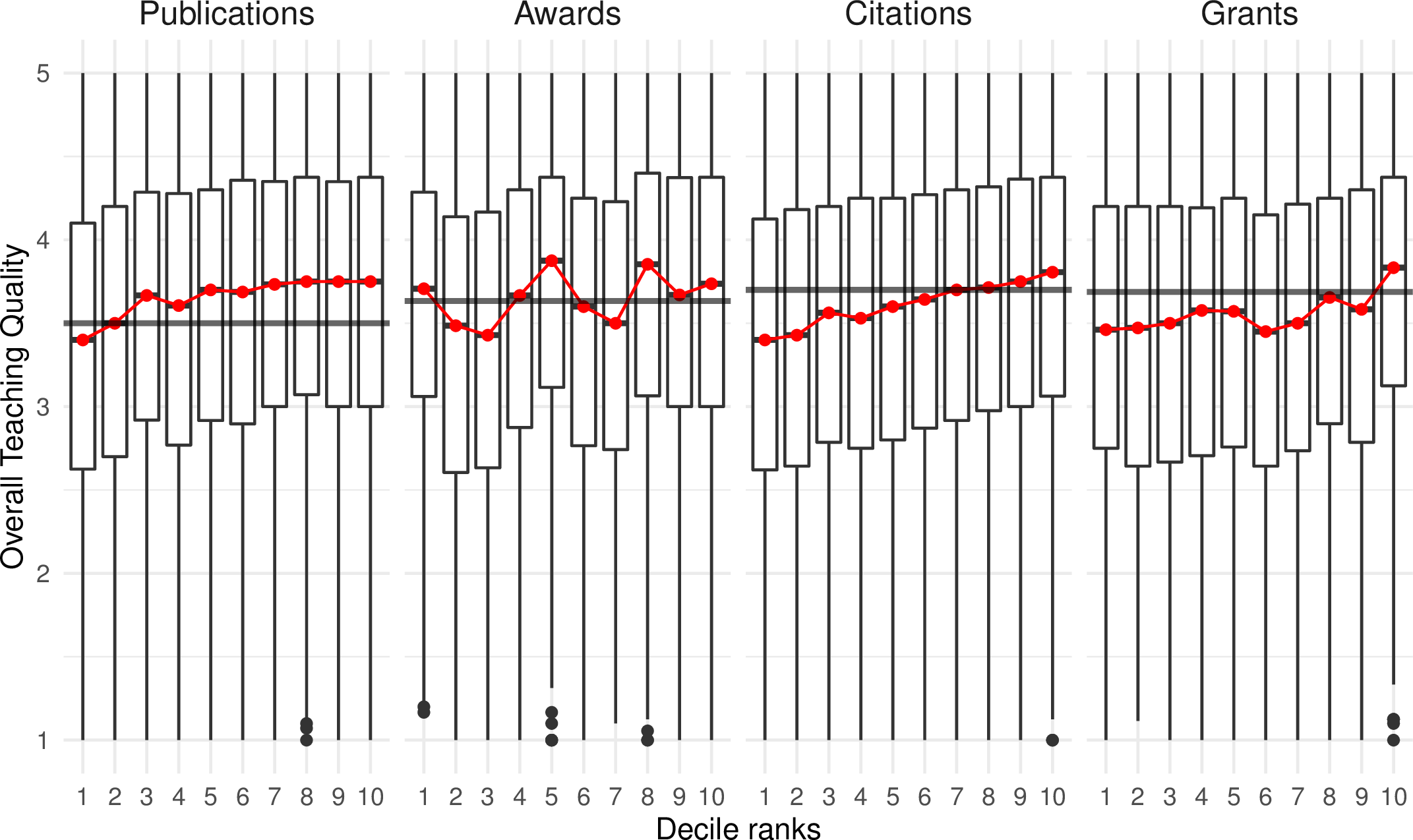

Supplement: S2 Fig — Boxplots of ratings of overall teaching quality for faculty having a positive non-zero value for field-normalized research indicators. Indicator performance is binned into deciles (x-axis). The horizontal grey line is the median for faculty with a value of zero in each indicator. The red line corresponds to the median rating of overall teaching quality for faculty in each decile bin. (TIF) [file pone.0233515.s003.tif]

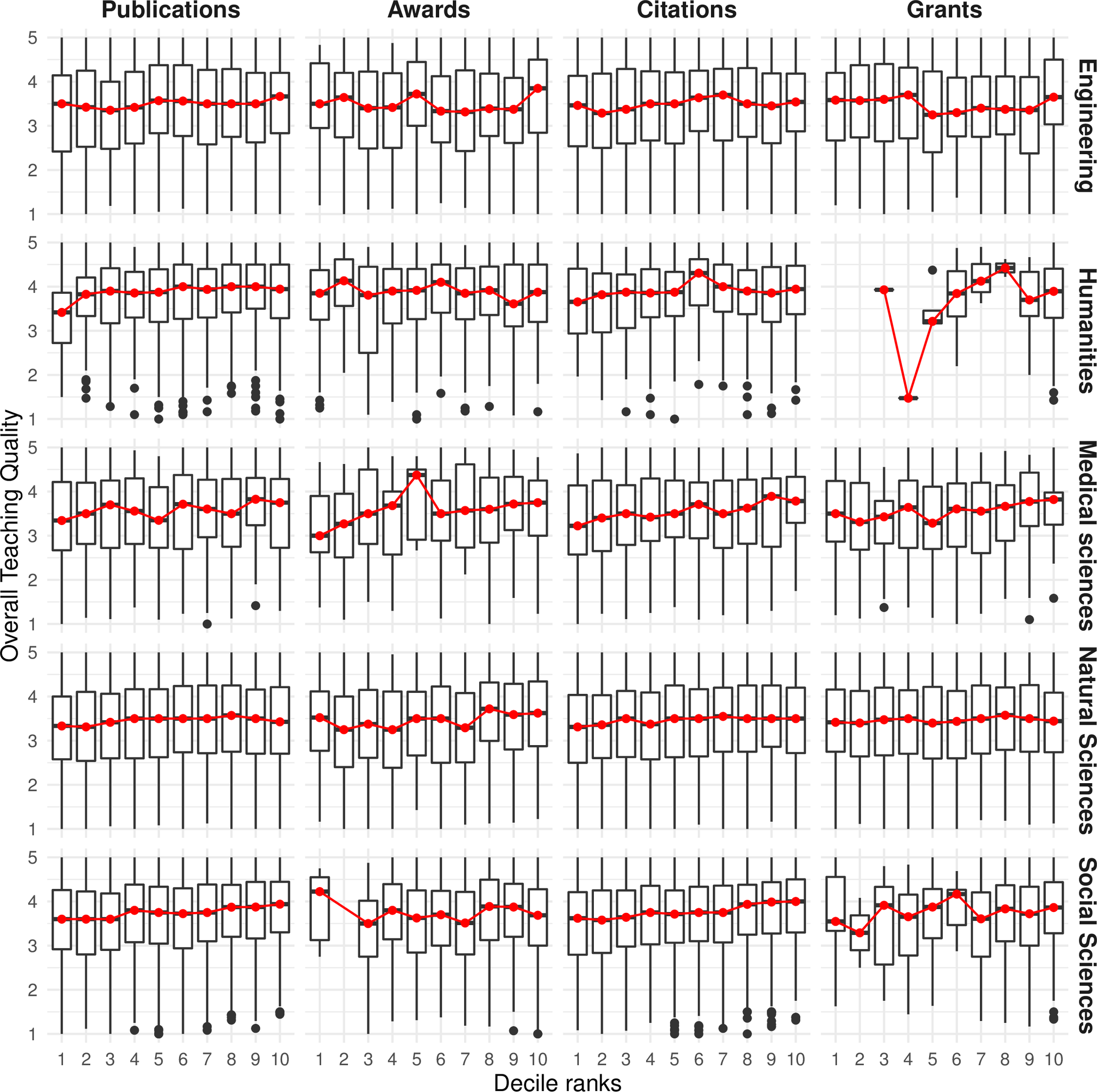

Supplement: S3 Fig — Boxplots of ratings of overall teaching quality for faculty having a positive non-zero value for field-normalized research indicators. Indicator performance is binned into deciles (x-axis), repeated for faculty in each of the five discipline categories. The red line corresponds to the median rating of overall teaching quality for faculty in each decile bin. (TIF) [file pone.0233515.s004.tif]

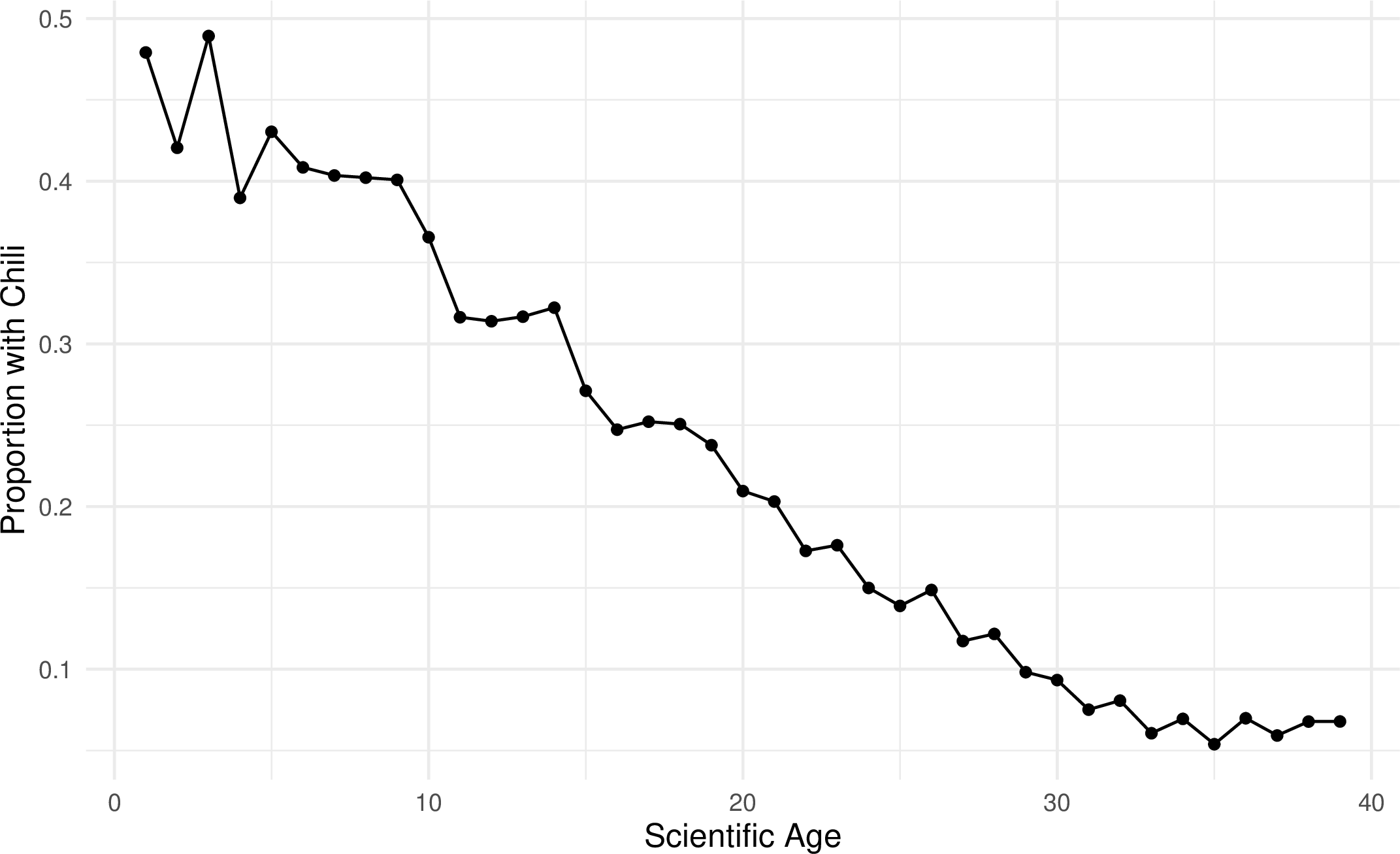

Supplement: S4 Fig — The proportion of faculty in the matched dataset that were assigned a chili pepper (y-axis), implicitly suggesting attractiveness, by scientific age (x-axis). (TIF) [file pone.0233515.s005.tif]

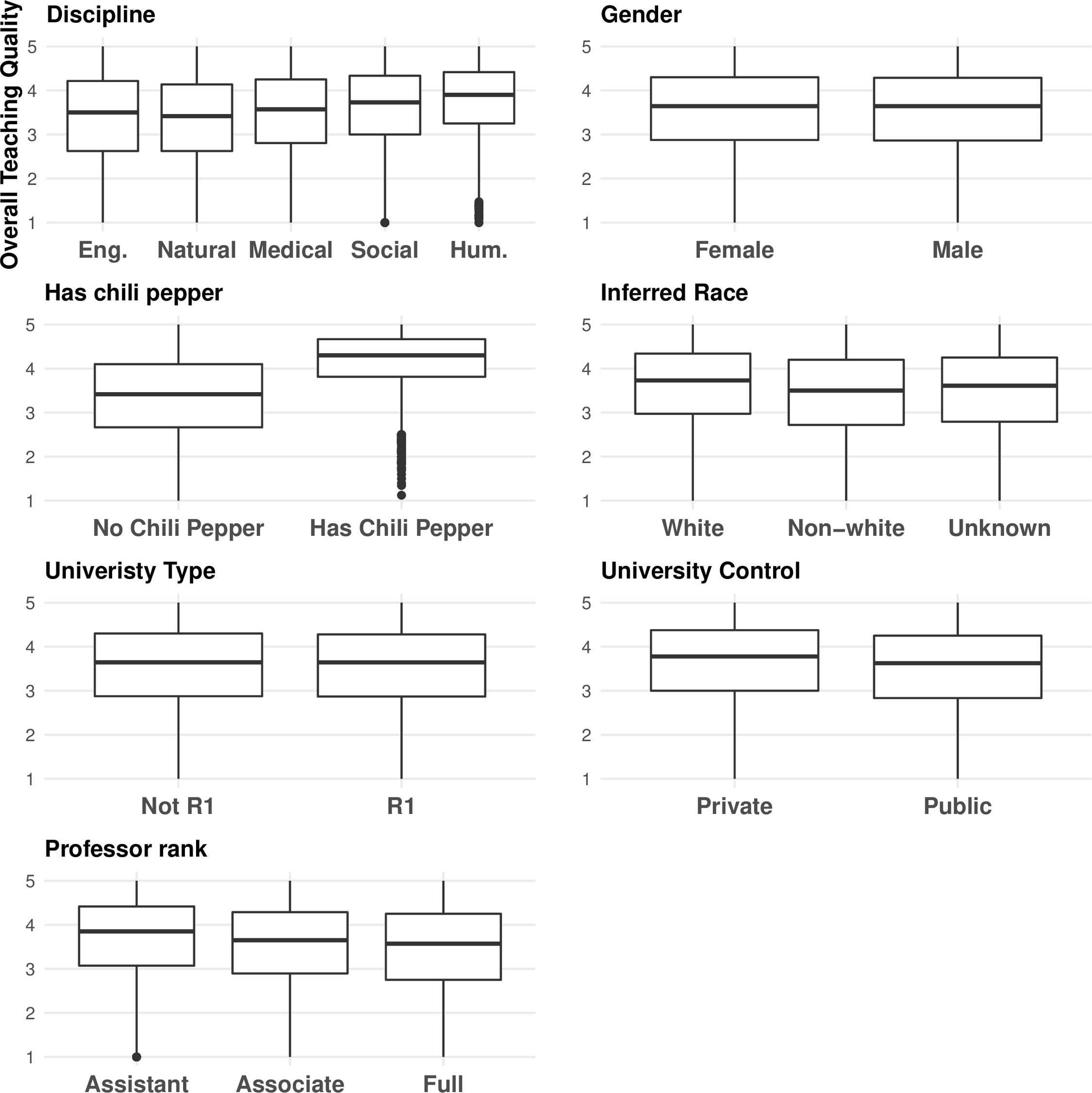

Supplement: S5 Fig — The distribution of ratings of overall teaching quality (y-axis) for values of each categorical variable (x-axis) from the matched dataset. Includes discipline, gender, whether the faculty has a chili pepper, inferred race, the university type, the university control, and the professor’s rank. (TIF) [file pone.0233515.s006.tif]

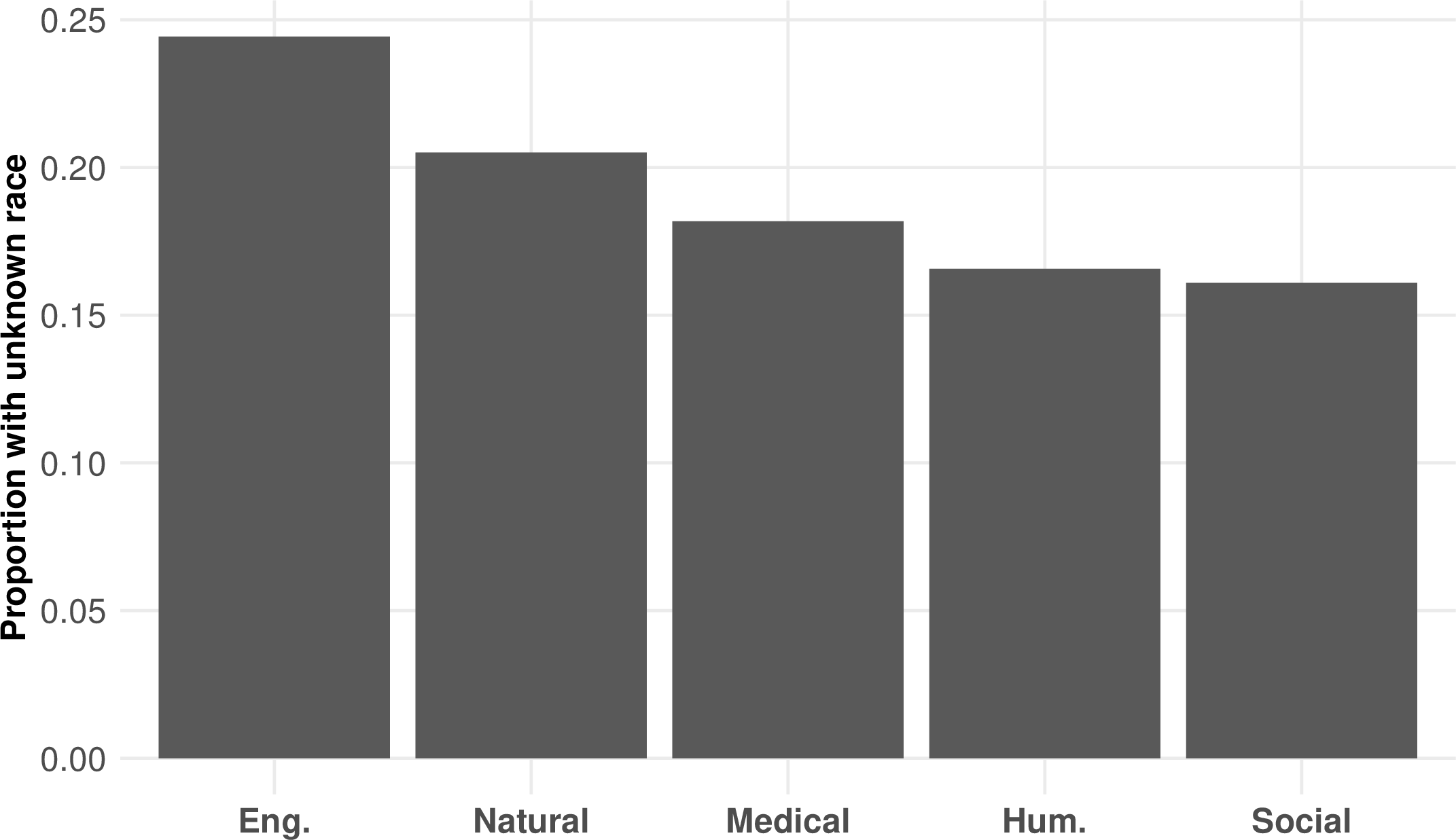

Supplement: S6 Fig — (TIF) [file pone.0233515.s007.tif]

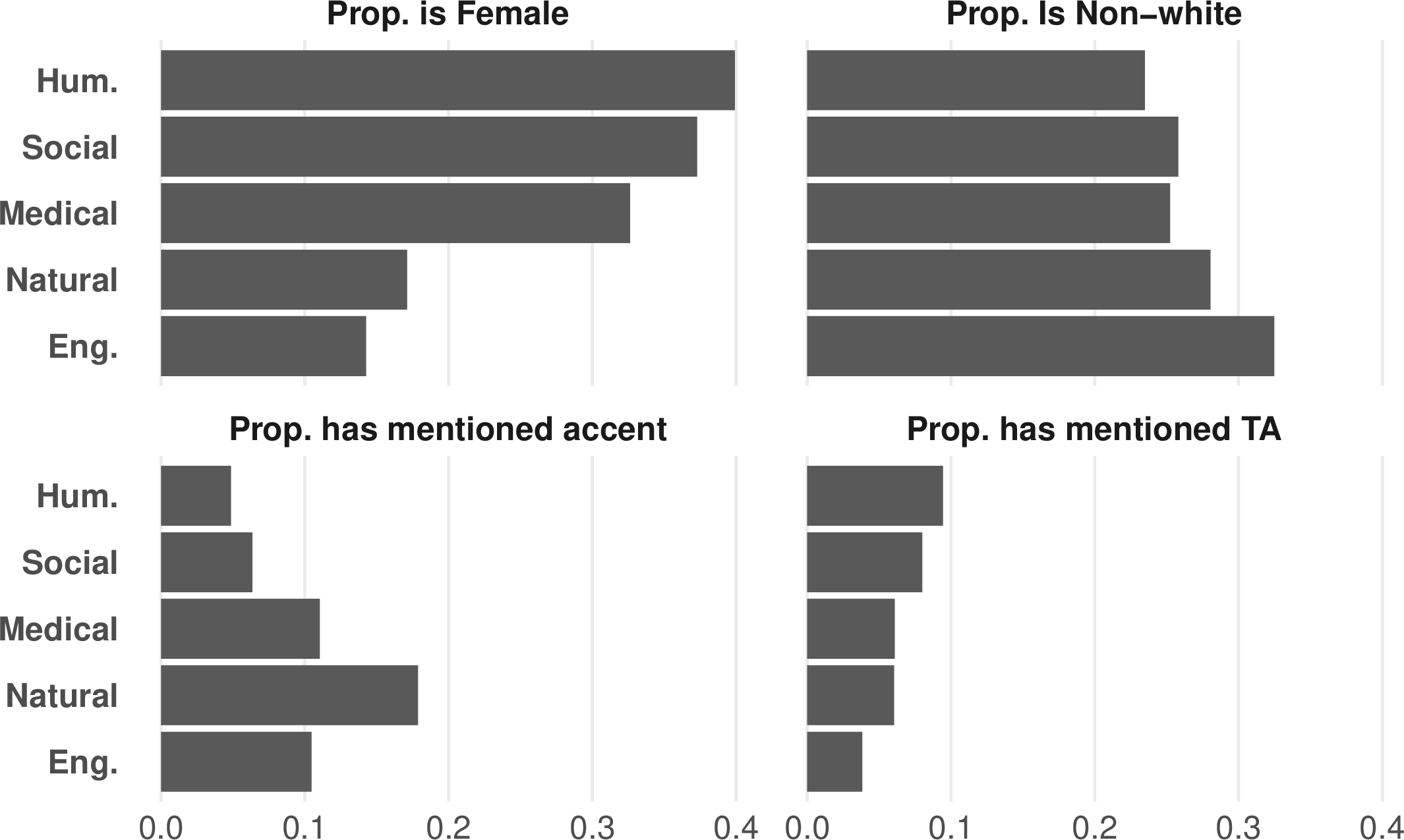

Supplement: S7 Fig — (TIF) [file pone.0233515.s008.tif]

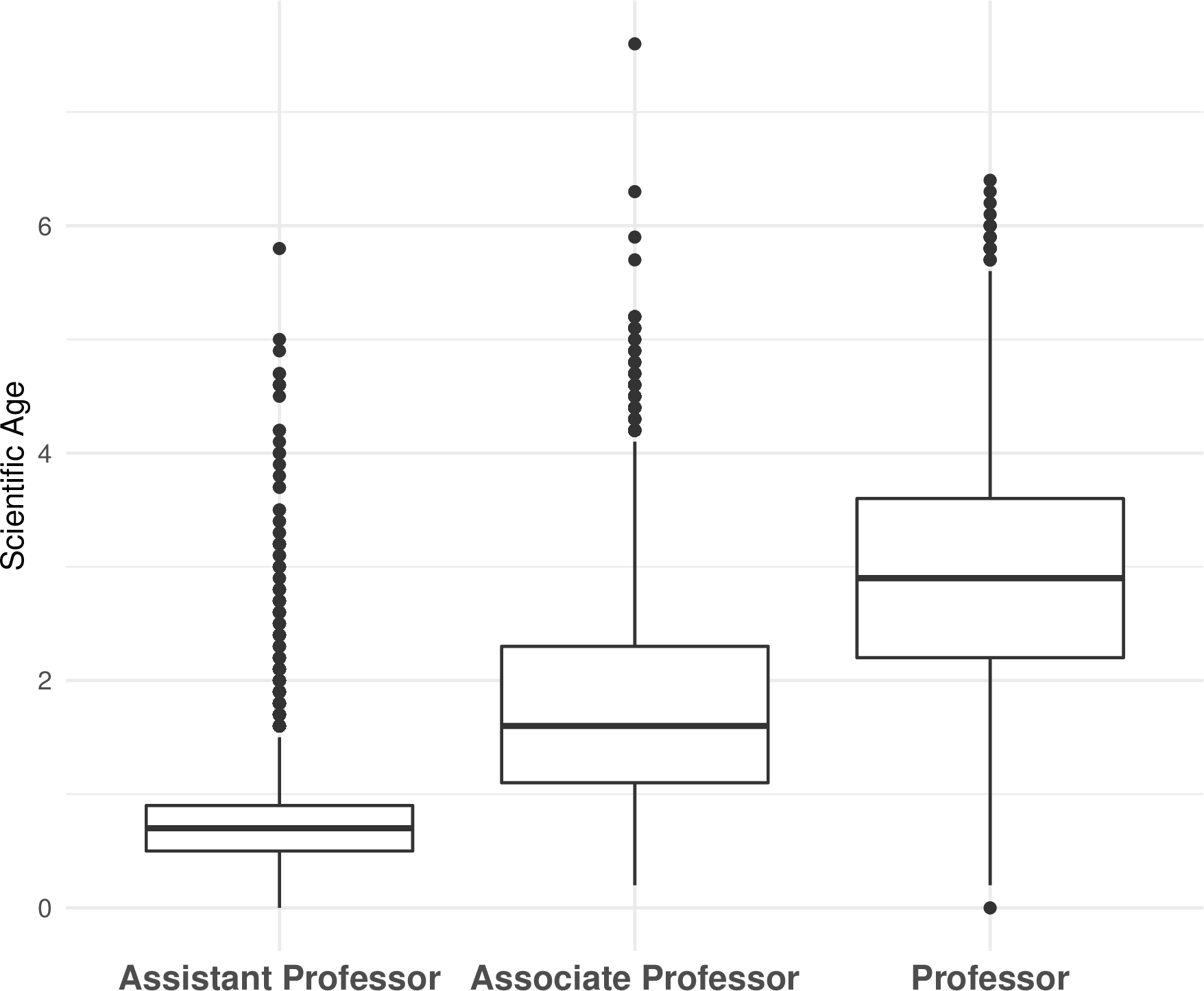

Supplement: S8 Fig — Boxplots for the distribution of scientific age (years since earning PhD or other terminal degree) and the rank of faculty, as indexed in AA2017. (TIF) [file pone.0233515.s009.tif]

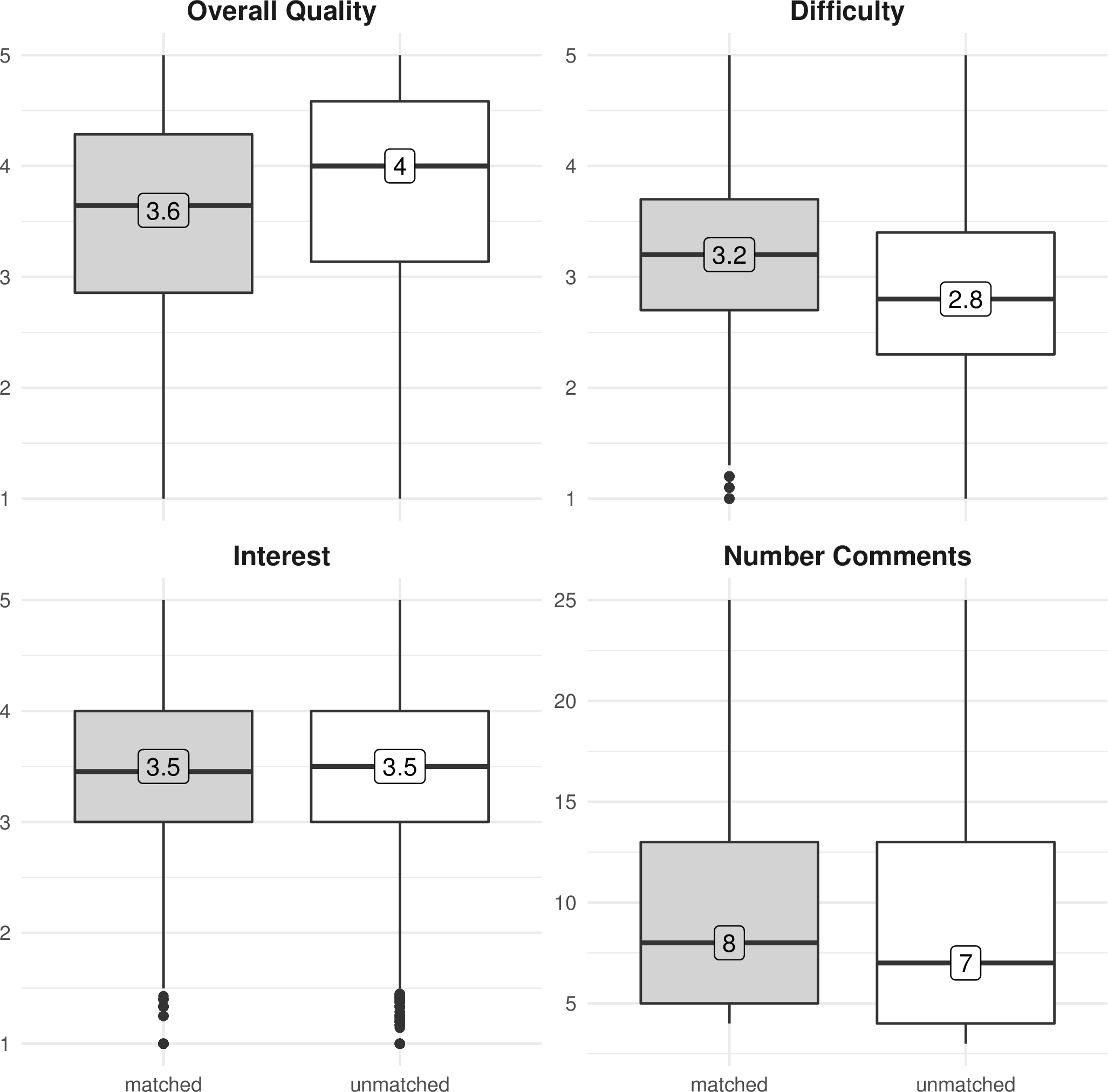

Supplement: S9 Fig — Boxplots detailing the distribution of overall quality, difficulty, interest, and the number of comments for individuals from RMP2018 were unmatched (white) vs. matched to records in AA2017 (dark grey). Labels in each boxplot state the median. (TIF) [file pone.0233515.s010.tif]
