# Supplementary material for: Exploring the personal and professional factors associated with student evaluations of tenure-track faculty
Source: PLoS One. 2020 Jun 3;15(6):e0233515. doi: 10.1371/journal.pone.0233515 (PMC7269236; doi:10.1371/journal.pone.0233515)
Supplement: S1 Table — (PDF) [file pone.0233515.s011.pdf]

Description of relevant variables from Academic Analytics 2016 dataset. Source indicates where the variable was collected by Academic Analytics. "Submitted" indicates that the variable was given to Academic Analytics by the institution.

Variable	Source	Description
Person name	Submitted; CrossRef, Institutional Websites	Full name of individual, as collected or submitted to Academic Analytics.
Institution Name	Submitted, Institutional Websites	Name of the institution in which the individual holds an affiliation
Degree Year	Submitted; Institutional Websites; CVs	Year in which the individual obtained their terminal degree (usually a doctorate)
Program Name	Submitted; Institutional websites	The name of the program to which the individual is affiliated, usually the name of their home department
Level 1 Name	Assigned by AA	Disciplinary classification of the individual, one of 172 low-level classifications created by AA and assigned based on the individuals' program affiliation
Article Count	CrossRef	Count of peer-reviewed journal articles published in 2013, 2014, 2015 and 2016. For coauthored articles, all authors are credited
Conf. Proceeding Count	CrossRef	Count of indexed conference proceedings published in 2013, 2014, 2015, and 2016. For coauthored articles, all authors are credited
Citation Count	CrossRef	Citations to articles and proceedings that were published in 2012, 2013, 2014, 2015, and 2016; data is derived from the CrossRef citation-linking network. Self-citations are included
Book Count	British Library, Baker & Taylor, and AA's internal collection efforts	Count of time appearing as author, co-author, editor, co-editor, and translator of books published in 2006-2016 (inclusive). Introductions, forewords, afterwards, and citations are not included in the 2014 Academic Analytics dataset. Due to their limited use of DOIs, chapters are limited.
Grant Count	Publicly available databases and FOIA requests	Count of grants data from 13 federal agencies and two non-federal sources matched to principal investigators. For NIH, NSF and NOAA grants, matching includes co-principal/multi-principal investigators
Award Count	AA's Internal collection efforts	The count of awards among honorific awards from 821 governing societies that are open to all people in a discipline, sub discipline, or a large subset of people at a national or international level, and that can be matched to individuals appearing in the Academic Analytics dataset
