# Supplementary material for: Exploring the personal and professional factors associated with student evaluations of tenure-track faculty
Source: PLoS One. 2020 Jun 3;15(6):e0233515. doi: 10.1371/journal.pone.0233515 (PMC7269236; doi:10.1371/journal.pone.0233515)
Supplement: S2 Table — (PDF) [file pone.0233515.s012.pdf]

Description of relevant variables extracted from RateMyProfessor.com reviews.

Variable	Description
Comment	An optional textual review of the teacher—in the newest iteration can contain up to 350 characters
Course	A code indicating the course that the student took with the teacher. Can be entered manually or selected from a list of options from a history of course codes used in previous reviews of the professor
Overall quality	A rating of the overall quality of the professor on an ordinal scale of 1 to 5. When posting a review on the RMP website the user is prompted with the question ”How would you rate this professor as an instructor?”. A rating of 1 indicates that the professor is poor quality while a rating of 5 indicates high quality.
Level of difficulty	A rating of the level of difficulty of the professor on an ordinal scale of 1 to 5. When posting a review a user is prompted with the question ”How hard did you have to work for this class?”. A rating of 1 indicates that the course is not difficult whereas a rating of 5 indicates that the course is very difficult.
Tags	The user is prompted to select at most three of a list of 23 predefined tags that describe the characteristics of the professor and their course
Interest	A legacy variable, this feature appears to have been removed at some point in the middle of 2017 and no longer features on reviews posted to the website. In older reviews this variable was intended to measure the interest that the student had in the subject of the course on a qualitative ordinal scale containing values of ”Low”, ”Meh”, ”Sorta Interested”, ”Really into It” and ”It’s My Life”
