# Supplementary material for: Exploring the personal and professional factors associated with student evaluations of tenure-track faculty
Source: PLoS One. 2020 Jun 3;15(6):e0233515. doi: 10.1371/journal.pone.0233515 (PMC7269236; doi:10.1371/journal.pone.0233515)
Supplement: S3 Table — (PDF) [file pone.0233515.s013.pdf]

Description of relevant variables extracted from RateMyProfessor.com teacher profiles.

**Variable**	**Description**
Name	Name of the teacher, as listed on the website, added by the users
Department	Department to which the teacher is affiliated as listed on the website and added by users
School	The School that the teacher is affiliated with, added by users
Overall Quality	An average of the individual ratings of a teacher's overall quality, intended as a general quality indicator. Individual ratings are on an ordinal scale between one and five, where one is considered poor quality, and five is considered high quality
Level of Difficulty	An average of the individual ratings of a teacher's level of difficulty, intended as an indicator of the difficulty of the teacher's courses. Individual ratings are on an ordinal scale between one and five, where one is considered easy and five is considered difficult
Chili	Presence of a "chili pepper" on the website, which indicates "hotness", or "attractiveness". When submitting a review, the user is asked to select between a positive and negative "hotness" rating. When the number of positive hotness ratings is greater than the number of negative hotness ratings, then that professor's profile is marked with a chili pepper
Tags	Individual reviewers can select from at most three of twenty pre-defined TAGS relating to characteristics of the professor or the course. These tags are then aggregated at the level of the professor. While the RMP website includes counts for the number of times these tags have been applied, we only capture a Boolean value indicating the presence of a tag
