# Supplementary material for: Exploring the personal and professional factors associated with student evaluations of tenure-track faculty
Source: PLoS One. 2020 Jun 3;15(6):e0233515. doi: 10.1371/journal.pone.0233515 (PMC7269236; doi:10.1371/journal.pone.0233515)
Supplement: S5 Table — (PDF) [file pone.0233515.s015.pdf]

**Results of Kendall Rank Tau.** Results of non-parametric test of dependence between the RMP2018 overall quality rating of the population, tested against each of the AA2017 research indicators.

| Tested Variable | Z-Value | Estimated Tau | P-Value |
|---|---|---|---|
| Article Count | 11.349 | 0.0453 | <2.2e-16 |
| Award Count | 2.1285 | 0.0093 | 0.0333 |
| Book Count | 10.122 | 0.0444 | <2.2e-16 |
| Citation Count | 3.2322 | 0.0130 | 0.00123 |
| Proceeding Count | -8.990 | -0.0411 | <2.2e-16 |
| Grant Count | -5.9901 | -0.0264 | 2.097e-09 |
