# Supplementary material for: Exploring the personal and professional factors associated with student evaluations of tenure-track faculty
Source: PLoS One. 2020 Jun 3;15(6):e0233515. doi: 10.1371/journal.pone.0233515 (PMC7269236; doi:10.1371/journal.pone.0233515)
Supplement: S6 Table — (PDF) [file pone.0233515.s016.pdf]

Results of multiple linear regression model with interactions. For binary variables, “false” is always used as the reference level. For Gender, “female” is used as the reference. For race, ”Non-White” is used as the reference. For rank, “Assistant” is used as the reference. For Discipline, “Engineering” is set as the reference. For Uni. Control, “Private” is used as the reference. For Uni. Type, “Not R1” is used as the reference. For all research indicators, “Low” is used as the reference. Estimates are listed first, followed by 95th percentile confidence intervals. Interactions are marked as variables separated by a ”*”.

	Dependent variable:
	overall
Is Male	0.133^{**} (0.027, 0.239)
Scientific Age	-0.124^{***} (−0.153, −0.095)
Mentions Accent = True	-0.161^{***} (−0.225, −0.097)
Has Chili Pepper	0.424^{***} (0.381, 0.467)
Rank = Associate	0.021 (−0.029, 0.071)
Rank = Full	0.131^{***} (0.065, 0.197)
Race unknown	0.017 (−0.035, 0.069)
Race Lilely White	0.082^{***} (0.041, 0.123)
Difficulty	-0.392^{***} (−0.402, −0.382)
Student Interest	0.329^{***} (0.319, 0.339)
Mentions TA = True	-0.183^{***} (−0.218, −0.148)
Citedness = Moderate	−0.023 (−0.053, 0.007)
Citedness = High	−0.034 (−0.086, 0.018)
Output = Moderate	0.035^{*} (−0.0003, 0.070)
Output = High	0.023 (−0.031, 0.078)
Grants Held = Moderate	−0.0003 (−0.025, 0.024)
Grants Held = High	0.032 (−0.023, 0.087)
Awards Won = Moderate	0.010 (−0.011, 0.030)
Awards Won = High	0.006 (−0.076, 0.087)
Humanities	0.292^{***} (0.206, 0.378)
Medical Sci.	0.182^{***} (0.079, 0.286)
Natural Sci.	0.140^{***} (0.054, 0.226)
Social Sci.	0.093^{**} (0.011, 0.176)
Uni. Type = R1	-0.031^{***} (−0.051, −0.010)
Uni. Control = Public	-0.086^{***} (−0.111, −0.060)
#Reviews	0.006^{***} (0.004, 0.007)
Is Male * Has Chili	−0.008 (−0.059, 0.044)
Is Male * Humanities	-0.145^{***} (−0.237, −0.052)
Is Male * Medical	−0.092 (−0.210, 0.026)
Is Male * Natural	-0.082^{*} (−0.175, 0.011)
Is Male * Social	−0.050 (−0.140, 0.041)
Is Male * Scientific Age	−0.011 (−0.043, 0.021)
Is Male * Race unknown	0.041 (−0.020, 0.103)
Is Male * Race Likely White	0.051^{**} (0.002, 0.100)
Is Male * Mentions Accent	−0.014 (−0.086, 0.059)
Is Male * Rank Associate	0.039 (−0.023, 0.100)
Is Male * Rank Full	0.011 (−0.066, 0.088)
Constant	3.140^{***} (3.039, 3.242)
Observations	18,973
R^2	0.515
Adjusted R^2	0.514
Residual Std. Error	0.643 (df = 18935)
F Statistic	543.120^{***} (df = 37; 18935) (p = 0.000)
Note:	$^{*}p<0.1$; $^{**}p<0.05$; $^{***}p<0.01$
