# Supplementary material for: Exploring the personal and professional factors associated with student evaluations of tenure-track faculty
Source: PLoS One. 2020 Jun 3;15(6):e0233515. doi: 10.1371/journal.pone.0233515 (PMC7269236; doi:10.1371/journal.pone.0233515)
Supplement: S7 Table — (PDF) [file pone.0233515.s017.pdf]

Results of multiple linear regression model using continuous research performance indicators. For binary variables, “false” is always used as the reference level. For Gender, “female” is used as the reference. For race, ”Non-White” is used as the reference. For “Rank”, “Assistant” is used as the reference. For Discipline, “Engineering” is set as the reference. For Uni. Control, “Private” is used as the reference. For Uni. Type, “Not R1” is used as the reference. Continuous field-normalized values are used for research performance indicators. Estimates are listed first, followed by 95th percentile confidence intervals.

	Dependent variable:
	overall
Is Male	0.105*** (0.083, 0.126)
Scientific Age	−0.133*** (−0.146, −0.120)
Mentions Accent = True	−0.173*** (−0.203, −0.143)
Has Chili Pepper	0.416*** (0.392, 0.441)
Rank = Associate	0.047*** (0.018, 0.077)
Rank = Full	0.135*** (0.099, 0.170)
Race Unknown	0.046*** (0.018, 0.073)
Race Likely White	0.118*** (0.095, 0.140)
Difficulty	−0.391*** (−0.400, −0.381)
Student Interest	0.329*** (0.319, 0.339)
Mentions TA = True	−0.184*** (−0.219, −0.148)
Citations	−0.004* (−0.008, 0.001)
Publications	0.008 (−0.002, 0.018)
Awards	0.006* (−0.001, 0.012)
Grants	−0.0001 (−0.003, 0.003)
Humanities	0.197*** (0.161, 0.233)
Medical Sci.	0.104*** (0.055, 0.152)
Natural Sci.	0.070*** (0.037, 0.103)
Social Sci.	0.046*** (0.011, 0.080)
Uni. Type = R1	−0.032*** (−0.052, −0.012)
Uni. Control = Public	−0.084*** (−0.109, −0.058)
#Reviews	0.006*** (0.004, 0.007)
Constant	3.179*** (3.126, 3.232)
Observations	18,973
R^2	0.514
Adjusted R^2	0.514
Residual Std. Error	0.643 (df = 18950)
F Statistic	911.970*** (df = 22; 18950) (p = 0.000)
Note:	*p<0.1; **p<0.05; ***p<0.01
