# Supplementary material for: Exploring the personal and professional factors associated with student evaluations of tenure-track faculty
Source: PLoS One. 2020 Jun 3;15(6):e0233515. doi: 10.1371/journal.pone.0233515 (PMC7269236; doi:10.1371/journal.pone.0233515)
Supplement: S8 Table — (PDF) [file pone.0233515.s018.pdf]

Little evidence of multicollinearity in discrete regression model. Generalized and adjusted variance inflation factor scores for the regression model of overall teaching quality with discrete research indicators.

	GVIF	Df	GVIF^(1/(2*Df))
Gender	1.115	1	1.056
Scientific Age	2.312	1	1.521
Mentions Accent	1.101	1	1.049
Has Chili Pepper	1.178	1	1.085
Rank	2.146	2	1.210
Race	1.067	2	1.016
Difficulty	1.121	1	1.059
Interest	1.121	1	1.059
Mentions TA	1.097	1	1.047
Citedness	2.766	2	1.290
Output	1.988	2	1.187
Grants	1.582	2	1.121
Awards Won	1.190	2	1.044
Discipline	2.251	4	1.107
Uni. Type	1.054	1	1.027
Uni. Control	1.019	1	1.010
Review Count	1.125	1	1.061
