# Supplementary material for: Exploring the personal and professional factors associated with student evaluations of tenure-track faculty
Source: PLoS One. 2020 Jun 3;15(6):e0233515. doi: 10.1371/journal.pone.0233515 (PMC7269236; doi:10.1371/journal.pone.0233515)
Supplement: S9 Table — (PDF) [file pone.0233515.s019.pdf]

Little evidence of multicollinearity in continuous regression model.
Generalized and adjusted variance inflation factor scores for the regression model of overall teaching quality with continuous research indicators.

	GVIF	Df	GVIF^(1/(2*Df))
Gender	1.115	1	1.056
Scientific Age	2.073	1	1.440
Mentions Accent	1.061	1	1.030
Has Chili Papper	1.175	1	1.084
Rank	2.029	2	1.193
Difficulty	1.119	1	1.058
Interest	1.120	1	1.058
Mentions TA	1.096	1	1.047
Norm. Citations	1.423	1	1.193
Norm. Publications	1.525	1	1.235
Norm. Awards	1.123	1	1.060
Norm. Grants	1.063	1	1.031
Discipline	1.200	4	1.023
Uni. Type	1.034	1	1.017
Uni. Control	1.019	1	1.010
Review Count	1.121	1	1.059
