# Supplementary material for: Exploring the personal and professional factors associated with student evaluations of tenure-track faculty
Source: PLoS One. 2020 Jun 3;15(6):e0233515. doi: 10.1371/journal.pone.0233515 (PMC7269236; doi:10.1371/journal.pone.0233515)
Supplement: S10 Table — (PDF) [file pone.0233515.s020.pdf]

Little difference between population of matched and unmatched Academic Analytics faculty. Shown are characteristics of the faculty in the matched dataset and of the tenure and tenure-track faculty of the unmatched Academic Analytics dataset.

Variable	Measure	Matched	Unmatched
Gender	% Male	71.5	64.2
	% Female	28.5	35.8
Inferred Race	% White	54.4	56.9
	% Non-White	26.9	25.5
	% Unknown	18.8	17.5
Professional Rank	%Assistant	17.8	23.4
	%Associate	35.9	27.3
	% Full	46.4	49.3
Scientific Age	1st Quartile	11	12
	Median	20	21
	3rd Quartile	30	21
University Type	% R1	69.9	39.7
	% Not R1	30.1	30.3
University Control	%Private	15.7	28.4
	% Public	84.3	71.6
Discipline	% Engineering	10.7	13.2
	% Social Science	21.9	25
	% Medical Science	5.5	26.7
	% Natural Science	33.6	22.9
	% Humanities	21.3	12.3
Publications	% No Publications	19.6	14.2
	% Moderate Publications	73.1	76.8
	% High Publications	7.2	9.5
Citations	% No Citations	28	19
	% Moderate Citations	66.2	72.7
	% High Citations	5.8	8.4
Grants	% No Grants	65.8	60.6
	% Moderate Grants	31.1	35.4
	% High Grants	3.1	4
Awards	% No Awards	64.6	67.5
	% Moderate Awards	33.9	28.9
	% High Awards	1.5	3.6
