# Supplementary material for: Exploring the personal and professional factors associated with student evaluations of tenure-track faculty
Source: PLoS One. 2020 Jun 3;15(6):e0233515. doi: 10.1371/journal.pone.0233515 (PMC7269236; doi:10.1371/journal.pone.0233515)
Supplement: S11 Table — (PDF) [file pone.0233515.s021.pdf]

Average counts of research item, by discipline

Discipline	Articles	Proceedings	Books	Awards	Citations	Grants
Engineering	12.034	5.921	0.348	0.805	189.504	1.566
Humanities	1.451	0.044	1.721	0.846	3.428	0.114
Medical Sciences	11.917	0.398	0.304	0.616	207.930	1.345
Natural Sciences	15.319	1.931	0.340	0.755	474.638	1.452
Social Sciences	5.854	0.182	0.990	0.489	62.174	0.357
